# Upregulation of the Adhesin Gene *EPA1* Mediated by *PDR1* in *Candida glabrata* Leads to Enhanced Host Colonization

**DOI:** 10.1128/mSphere.00065-15

**Published:** 2016-03-02

**Authors:** Luis A. Vale-Silva, Beat Moeckli, Riccardo Torelli, Brunella Posteraro, Maurizio Sanguinetti, Dominique Sanglard

**Affiliations:** a Institute of Microbiology, University of Lausanne and University Hospital Center, Lausanne, Switzerland; b Institute of Microbiology, Università Cattolica del Sacro Cuore, Rome, Italy; c Institute of Public Health (Section of Hygiene), Università Cattolica del Sacro Cuore, Rome, Italy; University of Texas Health Science Center

**Keywords:** *Candida*, drug resistance, adherence, fungus-host interactions

## Abstract

*Candida glabrata* is the second most common *Candida* species causing disseminated infection, after *C. albicans*. *C. glabrata* is intrinsically less susceptible to the widely used azole antifungal drugs and quickly develops secondary resistance. Resistance typically relies on drug efflux with transporters regulated by the transcription factor Pdr1. Gain-of-function (GOF) mutations in *PDR1* lead to a hyperactive state and thus efflux transporter upregulation. Our laboratory has characterized a collection of *C. glabrata* clinical isolates in which azole resistance was found to correlate with increased virulence *in vivo*. Contributing phenotypes were the evasion of adhesion and phagocytosis by macrophages and an increased adhesion to epithelial cells. These phenotypes were found to be dependent on *PDR1* GOF mutation and/or *C. glabrata* strain background. In the search for the molecular effectors, we found that *PDR1* hyperactivity leads to overexpression of specific cell wall adhesins of *C. glabrata*. Further study revealed that *EPA1* regulation, in particular, explained the increase in adherence to epithelial cells. Deleting *EPA1* eliminates the increase in adherence in an *in vitro* model of interaction with epithelial cells. In a murine model of urinary tract infection, *PDR1* hyperactivity conferred increased ability to colonize the bladder and kidneys in an *EPA1*-dependent way. In conclusion, this study establishes a relationship between *PDR1* and the regulation of cell wall adhesins, an important virulence attribute of *C. glabrata*. Furthermore, our data show that *PDR1* hyperactivity mediates increased adherence to host epithelial tissues both *in vitro* and *in vivo* through upregulation of the adhesin gene *EPA1*.

**IMPORTANCE** *Candida glabrata* is an important fungal pathogen in human diseases and is also rapidly acquiring drug resistance. Drug resistance can be mediated by the transcriptional activator *PDR1*, and this results in the upregulation of multidrug transporters. Intriguingly, this resistance mechanism is associated in *C. glabrata* with increased virulence in animal models and also with increased adherence to specific host cell types. The *C. glabrata* adhesin gene *EPA1* is a major contributor of virulence and adherence to host cells. Here, we show that *EPA1* expression is controlled by *PDR1* independently of subtelomeric silencing, a known *EPA1* regulation mechanism. Thus, a relationship exists between *PDR1*, *EPA1* expression, and adherence to host cells, which is critical for efficient virulence. Our results demonstrate that acquisition of drug resistance is beneficial for *C. glabrata* in fungus-host relationships. These findings further highlight the challenges of the therapeutic management of *C. glabrata* infections in human patients.

## Abstract (precis)

*Candida glabrata* is an important fungal pathogen in human diseases and is also rapidly acquiring drug resistance. Drug resistance can be mediated by the transcriptional activator *PDR1*, and this results in the upregulation of multidrug transporters. Intriguingly, this resistance mechanism is associated in *C. glabrata* with increased virulence in animal models and also with increased adherence to specific host cell types. The *C. glabrata* adhesin gene *EPA1* is a major contributor of virulence and adherence to host cells. Here, we show that *EPA1* expression is controlled by *PDR1* independently of subtelomeric silencing, a known *EPA1* regulation mechanism. Thus, a relationship exists between *PDR1*, *EPA1* expression, and adherence to host cells, which is critical for efficient virulence. Our results demonstrate that acquisition of drug resistance is beneficial for *C. glabrata* in fungus-host relationships. These findings further highlight the challenges of the therapeutic management of *C. glabrata* infections in human patients.

## INTRODUCTION

*Candida glabrata* is an opportunistic pathogen that is able to cause invasive infection in susceptible patients. Because *C. glabrata* is an opportunistic pathogen, host susceptibility is critical to initiate active infection, and over the last half century, the human population at risk has increased (1). This trend is based on an increased number of immunosuppressed patients. Several factors, such as the occurrence of different cancers, the use of organ transplants, the persistence of the AIDS pandemic, the use of broad-spectrum antibiotics, and the expansion of the elderly population, have contributed to the increase of this patient population (2). *C. glabrata* deploys several distinct factors to infect its host. *C. glabrata* is less pathogenic than *Candida albicans* (3); however, it is able to persist in experimental infection models for long periods by eliciting only mild immune responses (4, 5). An important virulence attribute of *C. glabrata* is its capacity to adhere to host tissues. The genome of *C. glabrata* contains a high number of genes (around 60 in the CBS138 genome) encoding predicted glycosylphosphatidylinositol (GPI)-anchored adhesin-like cell wall proteins (6). Adhesins of *C. glabrata* are distributed in several groups. The *EPA* (epithelial adhesion) subfamily is crucial for interaction with host cells. *EPA1* is the first member of this family and was identified by Cormack et al. (7) as a major mediator of adherence to epithelial cells. Interestingly, a majority of *C. glabrata* adhesin genes exhibit subtelomeric locations and are thus under the control of transcriptional silencing by chromatin-based and NAD-dependent regulation mechanisms (8). Since *C. glabrata* is a nicotinic acid (NA) auxotroph, NAD-limiting environments release adhesin genes from transcriptional repression when they have a subtelomeric location. Interestingly, the urinary tract is NAD poor and is thus a favorable host environment for adhesin expression. As a matter of fact, urinary tract infections (UTIs) caused by *C. glabrata* are very common (8).

In recent years, *C. glabrata* has emerged as an important fungal pathogen in many regions of the world (9). It is believed that antifungal drug usage and the emergence of antifungal drug-resistant *C. glabrata* isolates are partially responsible for this observation. Currently available antifungal agents for treatment of candidiasis are azoles, candins, and polyenes. Azoles and polyenes such as amphotericin interfere with sterols in these species at different levels. While azoles inhibit an important step in ergosterol biosynthesis (14α-lanosterol demethylation), amphotericin B forms a complex with ergosterol which compromises fungal viability. Candins target cell wall biosynthesis by inhibiting fungus-specific glucan synthesis. *C. glabrata* is intrinsically less susceptible to azole antifungals than is *C. albicans* (10). In addition, when exposed to this class of agents, *C. glabrata* rapidly develops resistance. It is believed that 20% of clinical isolates are azole resistant (11). Most azole-resistant isolates upregulate ATP-binding cassette (ABC) transporter genes, including *C. glabrata CDR1* (*CgCDR1*) and *CgCDR2*, which are important transporters for the development of azole resistance (12). This upregulation is mediated by *PDR1*, a transcriptional activator of the zinc cluster transcription factor family with a Zn(2)Cys(6) domain. Mutations in *PDR1* are called gain-of-function (GOF) mutations, since they are responsible for constitutive high expression of ABC transporters in resistant isolates. Multiple GOF mutations have been described, and they occur at different functional domains of the protein (12 – 15). *PDR1* interacts with the Mediator complex which bridges the activator and the transcriptional machinery in *C. glabrata* (16). Pdr1 regulates other genes in *C. glabrata* due to the presence of a pleiotropic drug resistance element (PDRE), a predicted Pdr1 binding target sequence, in the promoters of target genes (17). *PDR1* GOF mutations not only regulate ABC transporter genes but also target other genes responsible for other cellular processes. Strikingly, recent reports document increased proportions of multidrug-resistant *C. glabrata* isolates (i.e., isolates resistant to at least two different classes of agents) (18 – 20). It has been established that about 3 to 11% of azole-resistant isolates in the United States also exhibit candin resistance (21).

The selection of drug-resistant isolates by drug exposure can have a cost (or fitness cost) for the yeast. The cost of antifungal resistance is that strains may be less competitive than wild-type isolates, especially when the drug selection is removed. This potential decrease in fitness may indeed compromise their virulence. In *C. glabrata*, however, a few studies have addressed the relationship between acquisition of drug resistance and changes in virulence traits and found evidence of increased virulence. As reported by Poláková et al. (22) and Ahmad et al. (23), *C. glabrata* can form minichromosomes containing genes involved in drug resistance while colonizing the human host, which may selectively favor the expansion of this yeast *in vivo*. The effects of *PDR1* GOF mutations on *in vivo* fitness and on virulence have been addressed in *C. glabrata*. It was discovered that *PDR1* GOF mutations, in addition to their role as mediators of azole resistance, could enhance virulence of *C. glabrata* compared to wild-type isolates in mice (12). Enhanced virulence observed in mice was accompanied not only by elevated fungal loads in infected organ but also by treatment failure with azoles. Moreover, the change in virulence was paralleled with gain of *in vivo* fitness of strains carrying *PDR1* GOF mutations. The dogma that development of drug resistance is associated with fitness costs was therefore challenged by the results of Ferrari et al. (12), given that azole resistance had rather a fitness benefit *in vivo*. *CgCDR1*, the ABC transporter involved in azole resistance, and the open reading frame (ORF) CAGL0M12947g (*PUP1*, or *PDR1*-upregulated gene) are commonly upregulated by *PDR1* GOF mutations, and this effect was thought to explain enhanced virulence (24). While deletions of *CgCDR1* and *PUP1* decreased virulence of *C. glabrata* in a mouse model of infection, *PDR1*-independent overexpression resulted in intermediate virulence phenotypes. This suggested that other *C. glabrata* factors were responsible for the gain of virulence observed in drug-resistant isolates.

To gain insights about these additional factors, we further addressed the role of *PDR1* mutations in the interaction with several mammalian cell types, including murine bone marrow-derived macrophages (BMDMs), human acute monocytic leukemia cell line (THP-1)-derived macrophages, and different epithelial cell lines (25). We showed that, interestingly, *PDR1* GOF mutations led to decreased adherence to macrophages and to decreased uptake by the same cells. The interaction with epithelial cells revealed, however, an opposite trend. This suggested that *PDR1* GOF mutations may help *C. glabrata* to colonize epithelial host cells by increasing adherence to epithelial cell layers. These data highlighted that *PDR1* GOF mutations modulate the interaction with host cells with the consequence of increasing virulence.

Here, we show that specific adhesion attributes of *C. glabrata* are regulated by *PDR1*. Among them, the adhesin gene *EPA1* contributes to enhanced adhesion to epithelial mammalian cell lines when *PDR1* mutations are causing azole resistance. This *PDR1*-mediated effect may help this yeast species to be a more efficient pathogen.

## RESULTS

### Modulation of the interaction with host cells is *PDR1* GOF mutation and *C*. *glabrata* strain dependent.

**FIG 1:**
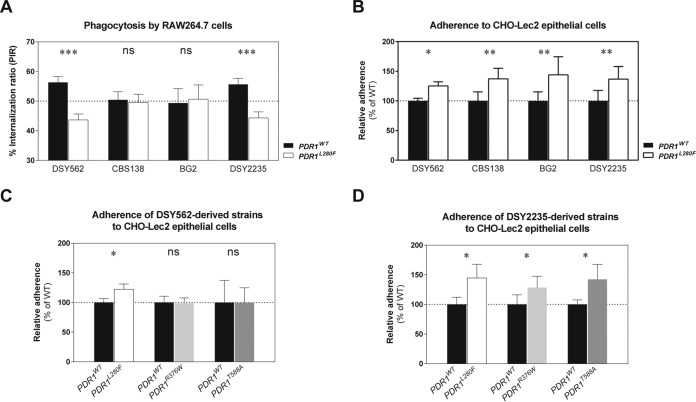
Influence of *PDR1* hyperactivity on the interaction of *C. glabrata* with mammalian host cells. Log-phase *C. glabrata* suspensions containing a 1:1 mix of two strains with either the *PDR1^WT^* or the *PDR1^L280F^* alleles were added to mammalian cell cultures. (A) Mixed yeast suspensions, labeled by expression of GFP or RFP, were added to preconfluent RAW 264.7 macrophage-like cells and incubated for 1 h at 37°C in a humid atmosphere with 5% CO_2_. The cultures were then washed to remove noninternalized yeasts, and percent internalization ratios (PIRs; percentage of each of the two yeast strains in the total number of yeasts) were determined. Test strains were constructed on four different genetic backgrounds: DSY562 (VSY103 to VSY106), CBS138 (VSY239 to VSY243), BG2 (VSY154, VSY155, VSY159, and VSY160), and DSY2235 (VSY231 to VSY234). (B) Mixed yeast suspensions of strains bearing the *PDR1^WT^* or the *PDR1^L280F^* alleles in different *C. glabrata* strain backgrounds were added to confluent CHO-Lec2 epithelial cell monolayers and incubated for 30 min at 37°C in a humid atmosphere with 5% CO_2_. The cultures were then washed four or five times to remove nonadherent yeasts, the epithelial cells were selectively lysed, and dilutions of the resulting yeast suspensions were plated on solid medium with or without fluconazole (to distinguish between azole-susceptible and azole-resistant yeast strains). Results are presented as CFU counts relative to the strain bearing the *PDR1^WT^* allele (set to 100%). Test strains were constructed on four different genetic backgrounds: DSY562 (VSY101 and VSY102), CBS138 (VSY236 and VSY237), BG2 (VSY149 and VSY150), and DSY2235 (VSY229 and VSY230). (C) Influence of two additional *PDR1* alleles on adherence of strain DSY562 (derived strains VSY101, VSY102, VSY134, and VSY135) to epithelial cells determined as described for the experiment in panel B. (D) Influence of two additional *PDR1* alleles on adherence of strain DSY2235 (derived strains VSY229, VSY230, VSY277, and VSY278) to epithelial cells determined as described for the experiment in panel B. Results are means ± standard deviations of a minimum of three independent experiments. Pairwise comparisons were performed using unpaired-sample two-tailed Mann-Whitney *U* tests. Bonferroni-adjusted *P* values of ≤0.05 were considered significant, and asterisks represent *P* value ranges (*, *P* value of <0.05; **, *P* value of <0.01; ***, *P* value of < 0.001). WT, wild type; ns, not significant.

In a previous report, we showed that *PDR1* hyperactivity in *C. glabrata* mediates evasion from phagocytosis by murine and human macrophages (25). This was reproduced across three different *PDR1* GOF mutations (L280F, R376W, and T588A) expressed in the azole-susceptible *C. glabrata* oropharyngeal clinical isolate DSY562. Surprisingly, we also found that the GOF mutation L280F led to increased adherence of DSY562 to three different epithelial cell lines (25). To follow up, we first addressed the reproducibility of these effects in different *C. glabrata* strain backgrounds, including the reference strain CBS138 (ATCC 2001), whose genome was sequenced and is publicly available (26); BG2, a well-known genetic background used in several studies (7, 8, 27, 28); and DSY2235, another azole-susceptible oropharyngeal clinical isolate from our strain collection (12). We introduced the same *PDR1* alleles (the wild-type *PDR1* allele from clinical isolate DSY562, *PDR1^WT^*, and the hyperactive allele from matched isolate DSY565, *PDR1^L280F^*) in these three additional *C. glabrata* strains and repeated competition phagocytosis assays using RAW 264.7 macrophage-like cells. Mixed suspensions of a given genetic background bearing either *PDR1^WT^* or *PDR1^L280F^* (and additionally expressing either green fluorescent protein [GFP] or red fluorescent protein [RFP], in order to distinguish between strains) were used to inoculate the macrophage cultures. Representative microscopy pictures are shown in Fig. S2 in the supplemental material. Our results confirmed the differences between DSY562 expressing the two alleles that were previously observed using primary murine macrophages and THP-1-derived human macrophages (Fig. 1A). However, this effect depended on the *C. glabrata* genetic background and was reproduced only by strain DSY2235 and not by CBS138 or BG2 (Fig. 1A). We next tested the adherence of the constructed strains to CHO-Lec2 epithelial cells and found that the increase in adherence was reproduced in all tested strain backgrounds (Fig. 1B). Our previous work showed that other tested *PDR1* alleles with GOF mutations R376W and T588A expressed in DSY562 reproduced the differences in the interaction with macrophages (25). Here, we addressed whether they also mediated increased adherence to CHO-Lec2 cells. Our results indicate that these GOF mutations could mediate a similar increase in adherence when expressed in DSY2235 (Fig. 1D) but not in DSY562 (Fig. 1C).

10.1128/mSphere.00065-15.1 Figure S1  Growth curves of DSY2235-based *C. glabrata* strains. The test strains were DSY2235 and *ura3*Δ isogenic strains on the DSY2235 background expressing either the *PDR1^WT^* or the *PDR1^L280F^* allele and a *URA3*-based episomal plasmid expressing *ScPGK1p-yEGFP*, *ScPGK1p-yEmRFP*, or the empty vector (Ø). The test strains were grown overnight and diluted to a density of 5.0 × 10^4^ cells/ml in selective medium (YNB minimal medium with appropriate amino acids and bases and without uracil). Cultures were grown under constant agitation at 30°C, and growth was determined by measuring the absorbance at 540 nm. Download Figure S1, TIF file, 0.8 MB. Copyright © 2016 Vale-Silva et al. 2016 Vale-Silva et al. This content is distributed under the terms of the Creative Commons Attribution 4.0 International license.

10.1128/mSphere.00065-15.2 Figure S2  Phagocytosis of *C. glabrata* by murine RAW 264.7 macrophage-like cells. Representative microscopy images acquired following 1 h of coincubation of *C. glabrata* strains with RAW 264.7 cells. Log-phase cultures of yeast strains labeled with GFP (VSY103) or RFP (VSY106) were mixed and added to preconfluent RAW 264.7 monolayers (established on top of round cover slides) at a multiplicity of infection of 1 for each of the two yeast strains (total multiplicity of infection, 2). Cocultures were stained with calcofluor white (CW), and the round cover slides were mounted on microscopy slides for visualization. The chitin-binding stain CW does not penetrate cells and thus stains noninternalized yeasts only. Pictures represent the differential interference contrast (DIC) view and a merge of green, red, and blue fluorescence images. Download Figure S2, TIF file, 2.3 MB. Copyright © 2016 Vale-Silva et al. 2016 Vale-Silva et al. This content is distributed under the terms of the Creative Commons Attribution 4.0 International license.

### *PDR1* hyperactivity leads to adhesin gene overexpression.

**FIG 2:**
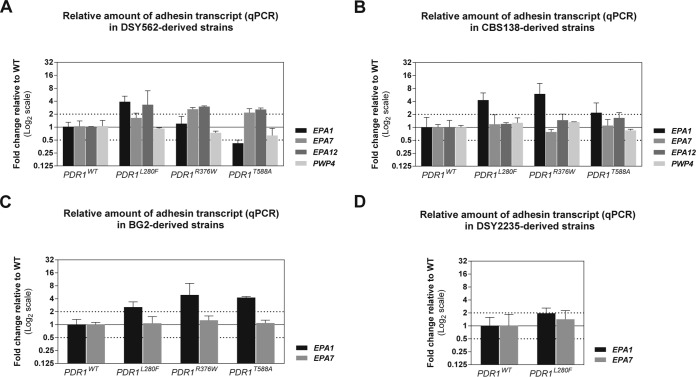
Influence of strain background and GOF mutations on *PDR1* on selected adhesin transcript levels. Whole-RNA extracts from log-phase *C. glabrata* cultures in rich medium were used as the templates to synthesize cDNA. Selected adhesin transcript levels were measured using qPCR with specific primers and TaqMan probes. Threshold cycles were normalized using *RDN5.8* quantification as the internal control. (A) Relative transcript levels of all four adhesins in DSY562 expressing different *PDR1* alleles (strains VSY43, VSY44, VSY132, and VSY133). (B) Relative transcript levels of all four adhesins in CBS138 expressing different *PDR1* alleles (strains VSY215, VSY216, VSY250, and VSY251). (C) Relative transcript levels of *EPA1* and *EPA7* in BG2 expressing different *PDR1* alleles (strains VSY144 to VSY147). (D) Relative transcript levels of *EPA1* and *EPA7* in DSY2235 expressing different *PDR1* alleles (strains VSY213 and VSY214).

In light of these *PDR1*-mediated changes in the interaction of *C. glabrata* with mammalian innate immune cells and epithelial cells, we reasoned that *PDR1* might regulate the expression of cell wall proteins with a role in adherence to host cells. We combined information from a previous genome-wide microarray study of *PDR1* hyperactivity in DSY562 performed in our laboratory (24), together with other transcriptomic studies in azole-resistant *C. glabrata* strains (14, 29, 30) and available functional knowledge on *C. glabrata* cell wall adhesins, in order to compile a small group of candidate genes for further testing. They included *EPA1*, *EPA7*, *EPA12*, and *PWP4*. We performed real-time quantitative PCR (qPCR) to test the impact of *PDR1* hyperactivity on the expression of these adhesins. Adhesin genes, particularly members of the *EPA* family, are generally difficult to target by qPCR due to the presence of tandem repeat sequence regions and a relatively high sequence similarity between different genes. We retrieved publicly available *C. glabrata* adhesin gene sequences from strains CBS138 and BG2 and sequenced *EPA1* from DSY562 and DSY2235. After qPCR optimization, efficient relative quantification of adhesin transcript levels could be achieved, including mutant and overexpression strains (illustrated for *EPA1* in Fig. S3 in the supplemental material). Our qPCR results revealed that the GOF mutation L280F mediated overexpression (at least 2-fold) of *EPA1* in all strain backgrounds (Fig. 2A to D). Other GOF mutations did not lead to *EPA1* overexpression in all strain backgrounds. Strikingly, comparison of transcript levels with adherence phenotypes for any given strain revealed a perfect correspondence between *EPA1* overexpression and increased adherence to epithelial cells (Fig. 1B to D and 2A to D). Transcript levels of *EPA7* and *EPA12* were either increased or not affected, with no obvious association with increased adherence to epithelial cells. *PWP4* expression was not affected by *PDR1* hyperactivity (Fig. 1B to D and 2A to D).

10.1128/mSphere.00065-15.3 Figure S3  Real-time quantitative PCR quantification of *EPA1* and *RDN5.8* transcript levels in *C. glabrata*. Whole-RNA extracts from log-phase *C. glabrata* cultures in rich medium were used as the templates to synthesize cDNA. *EPA1* transcript levels were measured using qPCR with specific primers and TaqMan probes. (A) Fluorescence change (*EPA1* TaqMan probe, background and passive dye corrected) over PCR cycle for nondiluted and three successive 10-fold-diluted cDNA samples of strain DSY562. (B) Fluorescence change (*RDN5.8* TaqMan probe, background and passive dye corrected) over PCR cycle for nondiluted and three successive 10-fold-diluted cDNA samples of strain DSY562. (C) Standard curves of mean threshold cycle (*C_T_*) over cDNA dilution for *EPA1* and *RDN5.8* of strain DSY562. *R*^2^ values for each independent experiment (rather than means of experiments) were >0.99. (D) Fluorescence change (*EPA1* TaqMan probe, background and passive dye corrected) over PCR cycle for different strains constructed on the DSY562 strain background (strains VSY43, VSY44, VSY166, and VSY220) and a blank control with no template cDNA. All data points and errors represent means ± standard deviations from a minimum of two independent experiments. Download Figure S3, TIF file, 1.1 MB. Copyright © 2016 Vale-Silva et al. 2016 Vale-Silva et al. This content is distributed under the terms of the Creative Commons Attribution 4.0 International license.

### Pdr1 regulates *EPA1* expression.

**FIG 3:**
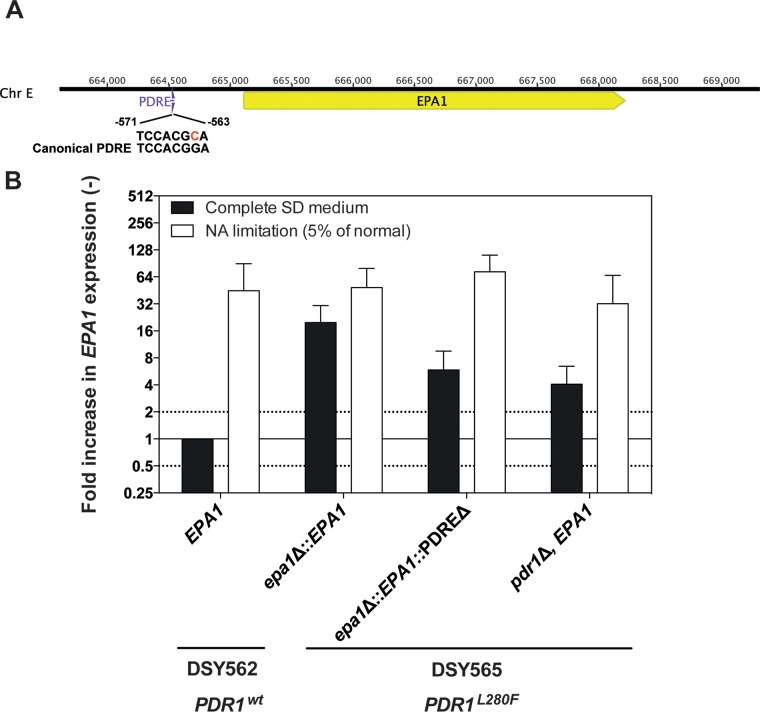
Regulation of *EPA1* by Pdr1. (A) Schematic illustration of the *EPA1* genomic region and localization of the PDRE-like sequence (TCCACGCA) positioned 563 bp upstream of the *EPA1* promoter matching the *PDR1* canonical binding site (TCCACGGA). Coordinates of chromosome E (Chr E) are indicated. (B) Deletion of the PDRE in *EPA1* and its effect on *EPA1* expression. *C. glabrata* isolates (VSY21, VSY265, VSY299, and VSY120) were grown with or without NA limitation as indicated, and *EPA1* gene expression was measured with biological triplicates. The strain lacking the PDRE in the genetic background of DSY565 (hyperactive *PDR1^L280F^*; VSY299) was subjected to the same treatment.

The association of *EPA1* overexpression with increased adherence to epithelial cells in *C. glabrata* strains with *PDR1* GOF mutations first indicated a role for *EPA1* as a Pdr1-mediated effector of the adherence phenotypes. Our results showed that *PDR1* regulates the expression of *EPA1*, but the question remains, however, whether this molecular regulation is direct or indirect. Interestingly, we noticed the presence of a putative PDRE in the promoter of *EPA1* (TCCACGCA, position starting −571 upstream of the *EPA1* start codon [Fig. 3A]) (30) matching the *PDR1* binding site (TCCACGGA) (17). The deletion of 72 bp centered on the putative PDRE in the *EPA1* promoter in a DSY562-derived strain containing the hyperactive *PDR1^L280F^* allele led to expression of *EPA1* being decreased by approximately 4-fold compared to a wild-type *EPA1* promoter (Fig. 3B). Interestingly, data published by Paul et al. (17) reporting the genomic binding sites of Pdr1 suggest that the *EPA1* promoter is targeted by Pdr1 (see Fig. S4A in the supplemental material). Chromatin immunoprecipitation (ChIP) with anti-Pdr1 on *EPA1* showed, however, that Pdr1 does not bind tightly the *EPA1* promoter (see Fig. S4B), thus raising the possibility of an indirect effect of Pdr1 on *EPA1*.

10.1128/mSphere.00065-15.4 Figure S4  Probing the binding of *PDR1* to the *EPA1* promoter. (A) Schematic representation of chromatin immunoprecipitation (ChIP) sequencing data from the work of Paul et al. (17) in the *EPA1* genomic region (nucleotide numbering according to CBS138 genome data is given). The alignment was produced with the CLC Genomics Workbench software and with data available at the Gene Expression Omnibus site (accession no. GSE59839). The yellow arrow represents the *EPA1* ORF and is drawn to scale. The arrow shows the position of a sequence (TCCACGCA) positioned 563 bp upstream of the *EPA1* promoter matching the Pdr1 binding site (TCCACGGA) (17). Reads that aligned with the genome are represented by blue bell shapes. (B) ChIP of *EPA1* and *CgCDR1* with Pdr1 antibody. Cells (strains SFY93, SFY114, and SFY115) were grown in 5% or 100% niacin, as indicated, to induce derepression of *EPA1* as described by Domergue et al. (8). PCR products were separated by gel electrophoresis, and size standards are indicated. ChIP was performed according to procedures published by Paul et al. (13, 17) and used the polyclonal antibody raised in rabbit against the DNA binding domain of Pdr1. ChIP PCRs on *EPA1* and *CgCDR1* were performed with primers EPA1a-ChIP (5′-ACTATCAAAGTACCAGATCCACTC-3′) and EPA1b-ChIP (5′-TGAAGGTTTCTCTTGGTTATTTTACTG-3′) and primers CgCDR1a-ChIP (5′-TCTTCTAGCAGCTATGAGTTGAGGAAG-3′) and CgCDR1b-ChIP (5′-CCATTGCCTCTACAGCATGCTTG-3′), respectively. Download Figure S4, TIF file, 2.3 MB. Copyright © 2016 Vale-Silva et al. 2016 Vale-Silva et al. This content is distributed under the terms of the Creative Commons Attribution 4.0 International license.

### *PDR1* hyperactivity-mediated increase in adherence to epithelial cells is dependent on *EPA1.*

**FIG 4:**
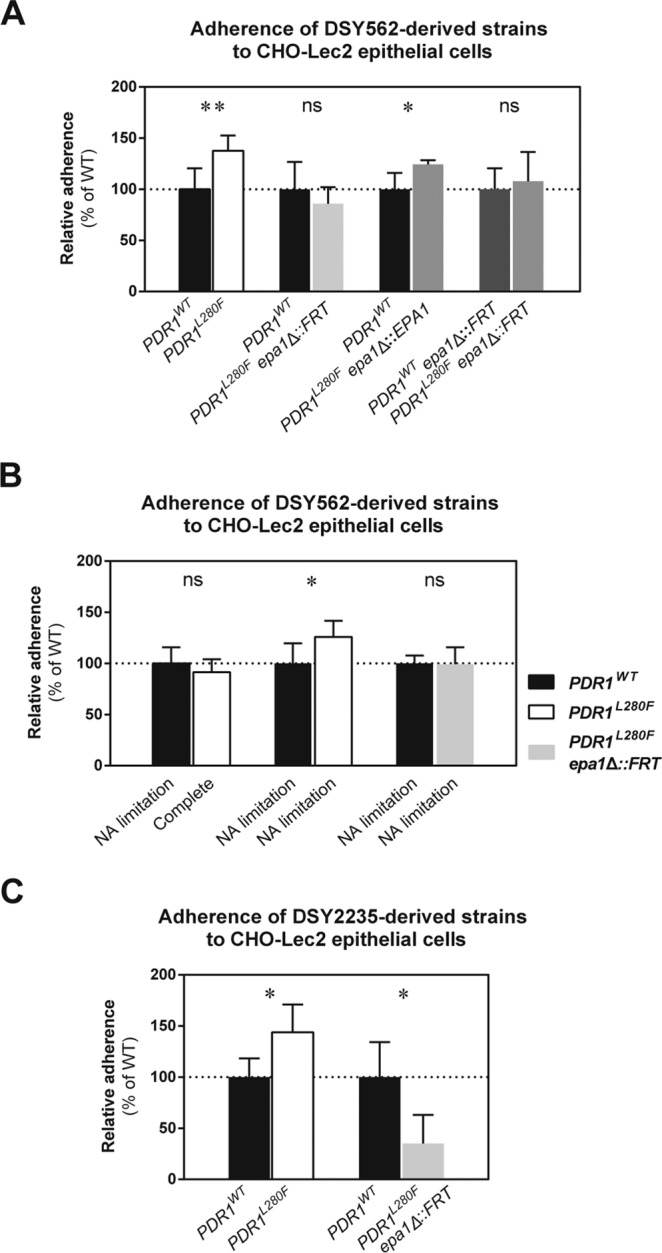
Influence of *PDR1* hyperactivity and *EPA1* on the interaction of *C. glabrata* with epithelial cells. Confluent CHO-Lec2 epithelial cell monolayers were infected with suspensions containing a 1:1 mix of log-phase *C. glabrata* strains with either the *PDR1^WT^* or the *PDR1^L280F^* allele in the presence or absence of *EPA1*. The cocultures were incubated for 30 min at 37°C in a humid atmosphere with 5% CO_2_ and then washed to remove nonadherent yeasts. The epithelial cells were selectively lysed, and dilutions of the resulting yeast suspensions were plated on solid medium with or without fluconazole (to distinguish between azole-susceptible and azole-resistant yeast strains). Results are presented as CFU counts relative to the strain bearing the *PDR1^WT^* allele (set to 100%). (A) Adherence of DSY562-derived strains (VSY101, VSY102, VSY244, and VSY266). (B) Adherence of DSY562-derived strains (VSY101, VSY102, and VSY293) grown either in complete medium or under adhesion-inducing NA limitation. (C) Adherence of DSY2235-derived strains (VSY229, VSY230, and VSY287). Results are means ± standard deviations of a minimum of three independent experiments. Pairwise comparisons were performed using unpaired-sample two-tailed Mann-Whitney *U* tests. Bonferroni-adjusted *P* values of ≤0.05 were considered significant, and asterisks represent *P* value ranges (*, *P* value of <0.05; **, *P* value of <0.01). WT, wild type; ns, not significant.

The data described above hint at a role for *EPA1* in the *PDR1*-mediated modulation of the interaction of *C. glabrata* with host epithelial tissues. In order to confirm this hypothesis, we inactivated *EPA1* in DSY562-derived strains and repeated the competition assays for adherence to epithelial CHO-Lec2 cells. As shown in Fig. 4A, the absence of *EPA1* in DSY562 with a *PDR1^L280F^* allele eliminated the increased adherence compared to the strain harboring a wild-type *PDR1* allele. This *epa1*Δ strain showed no difference in adherence to epithelial cells from the strain harboring the wild-type *EPA1* and *PDR1* alleles. Reversion of *EPA1* rescued the increase in adherence (Fig. 4A). *C. glabrata* is often found causing infection in nicotinic acid (NA)-deficient host environments (notably in the urinary tract). This host niche is known to lead to derepression of *EPA1* and other subtelomeric adhesin genes (8), thus signifying that *EPA1* can be upregulated even in the absence of *PDR1* GOF mutations. We recapitulated this situation by addressing adherence of *C. glabrata* grown either in complete medium or in NA-limited medium (5% of the regular NA concentration). When the wild type was grown under NA limitation, i.e., *PDR1*-independent adhesin expression-inducing conditions (as shown by qPCR for *EPA1* [see Fig. S5 in the supplemental material]), adherence to epithelial cells matched that of the strain with the hyperactive *PDR1^L280F^* allele grown under a normal NA concentration (Fig. 4B). Nevertheless, the increased adherence mediated by *PDR1* hyperactivity was still detected when both strains were grown under NA limitation (Fig. 4B). Taken together, our data confirm that *EPA1* mediates the difference in adherence between *C. glabrata* strains with wild-type and hyperactive *PDR1* alleles.

10.1128/mSphere.00065-15.5 Figure S5  Influence of growth under nicotinic acid (NA) limitation on *EPA1* expression. DSY562-based strains (SFY93, SFY114, and SFY115) were grown either in complete medium or under adhesion-inducing NA limitation (5% of the normal concentration). Whole-RNA extracts from log-phase *C. glabrata* cultures in rich medium were used as the templates to synthesize cDNA. *EPA1* transcript levels were measured using qPCR with specific primers and TaqMan probe. Threshold cycles were normalized using *RDN5.8* quantification as the internal control. Download Figure S5, TIF file, 0.8 MB. Copyright © 2016 Vale-Silva et al. 2016 Vale-Silva et al. This content is distributed under the terms of the Creative Commons Attribution 4.0 International license.

### Impact of *EPA1* on adherence to epithelial cells is *C*. *glabrata* strain dependent.

**FIG 5:**
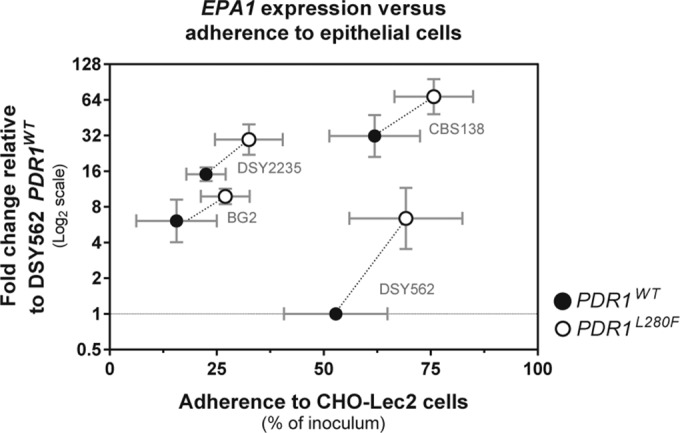
Comparison of the influence of *PDR1* hyperactivity on *EPA1* expression and the interaction of *C. glabrata* with epithelial cells in different strain backgrounds. Experiments were performed as detailed above in Fig. 1 and 2, with the same *C. glabrata* strains. The horizontal axis shows CFU counts as percentages of the inoculated CFU adhering to CHO-Lec2 epithelial cell monolayer experiments. The vertical axis shows *EPA1* transcript levels relative to DSY562 expressing the *PDR1^WT^* allele. Results are means ± standard deviations of a minimum of three independent experiments.

Inactivation of *EPA1* in DSY562 with the hyperactive *PDR1^L280F^* allele had an unexpectedly low impact on adherence to epithelial cells (reduction of adherence by less than 30%), thus making it almost indistinguishable from the wild type. Inactivating *EPA1* in DSY562 had a similar low impact on adherence (Fig. 4A). This suggests that *EPA1* was not required to maintain basal adherence levels in this strain. This is in contrast to the large adherence reduction (>90%) reported previously in BG2 (7). We tested the BG2 *epa1*Δ strain and observed the same large reduction in adherence (data not shown), thus ruling out inappropriate test conditions. This suggests an inherently low contribution of Epa1 for basal adherence of DSY562 to epithelial cells. We then inactivated *EPA1*, tested the adherence of DSY2235 containing the hyperactive *PDR1^L280F^* allele, and compared the results to the wild type. Adherence of the DSY2235 *epa1*Δ mutant dropped by more than 75% compared to the wild type (Fig. 4C), reminiscent of observations in BG2. While performing these experiments, we noticed that basal *EPA1* expression levels were highly variable between different *C. glabrata* strain backgrounds. Interestingly, these basal expression levels were proportional to the degree of adherence to epithelial cells. However, our results indicated that DSY562 stands as an exception. Even if this yeast expresses *EPA1* at the lowest levels among the tested isolates of this study, it was still second in terms of adherence to epithelial cells (Fig. 5). This, together with the low impact of *EPA1* on adherence, suggests that, unlike other *C. glabrata* strains (which rely heavily on Epa1 for adherence to epithelial cells), the DSY562 strain background relies primarily on alternative adhesins for basal adherence. Nevertheless, and even if *PDR1* hyperactivity mediates upregulation of additional adhesins in DSY562, *PDR1* hyperactivity still mediates increased adherence to epithelial cells through *EPA1* upregulation in this strain background.

### *PDR1* hyperactivity mediates an *EPA1*-dependent increase in colonization of the murine urinary tract.

**FIG 6:**
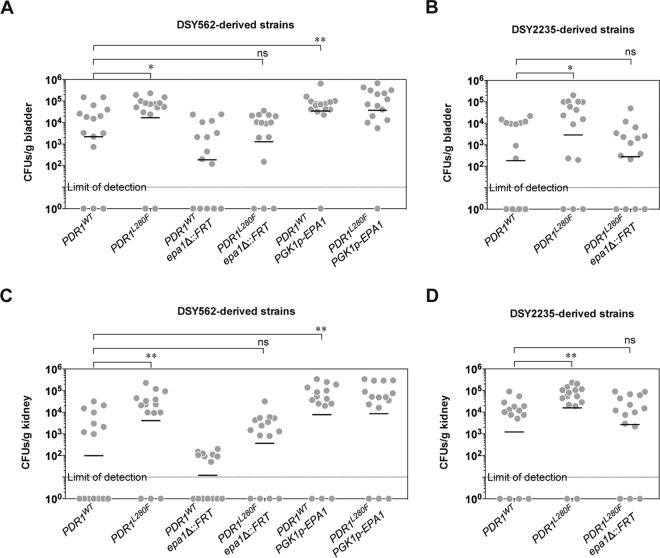
Influence of *PDR1* hyperactivity and *EPA1* on the colonization of the bladder and kidney by *C. glabrata* in the murine urinary tract infection (UTI) model. Experimental details are given in Materials and Methods. *C. glabrata* burdens in bladders and kidneys are presented as CFU/gram of organ (bars are geometric means). (A) Bladder burdens of DSY562-derived strains: SFY114, SFY115 (results confirmed using DSY562 and DSY565; data not shown), VSY271, VSY272, VSY220, and VSY221. (B) Bladder burdens of DSY2235-derived strains: VSY229, VSY230 (results confirmed using VSY213 and VSY214; data not shown), and VSY287. (C) Kidney burdens of DSY562-derived strains (same strains as in panel A). (D) Kidney burdens of DSY2235-derived strains (same strains as in panel B). Pairwise comparisons were performed using unpaired-sample two-tailed Mann-Whitney *U* tests. Bonferroni-adjusted *P* values of ≤0.05 were considered significant, and asterisks represent *P* value ranges (*, *P* value of <0.05; **, *P* value of <0.01). ns, not significant.

In order to determine the biological significance of *PDR1*-mediated regulation of *EPA1* expression, we employed a murine model of urinary tract infection (UTI). Besides strains expressing either *PDR1^WT^* or *PDR1^L280F^* and *epa1*Δ strains, we also addressed the impact of *PDR1*-independent *EPA1* overexpression. To this end, we constructed DSY562-derived strains expressing *EPA1* from an episomal plasmid under the control of a strong constitutive promoter (the *Saccharomyces cerevisiae* PGK1 [*ScPGK1*] promoter). The resulting strains displayed a strong increase in *EPA1* transcript levels, irrespective of the *PDR1* allele (see Fig. S6A in the supplemental material). This was accompanied by an increase in the measured adherence to epithelial cells to close to 100% of the inoculum under our test conditions (see Fig. S6B). We tested all these genetic variants from the DSY562 strain background in the UTI model, as well as a subset of them in the DSY2235 strain background. The results illustrate an increased ability of strains expressing *PDR1^L280F^* to colonize the bladder and then to reach the kidneys of infected mice compared to strains with *PDR1^WT^* (Fig. 6A to D). Just as in the case of adherence to epithelial cells, these phenotypes depended on *EPA1*, since *PDR1^L280F^* epa1Δ strains showed CFU counts indistinguishable from those of the *PDR1^WT^* strains in both DSY562 and DSY2235. The *ScPGK1* promoter-driven *EPA1* overexpression in DSY562 led to the highest observed CFU counts, with no statistically significant difference between *PDR1* alleles (Fig. 6A and C). With regard to *epa1*Δ mutants in DSY562 with *PDR1^L280F^*, even if they show a decrease in tissue burden in infected animals, they still show a higher burden than the *epa1*Δ *PDR1^WT^* strain. This suggests an additional, *EPA1*-independent effect of *PDR1* hyperactivity in the colonization of host urinary tract organs by *C. glabrata*.

10.1128/mSphere.00065-15.6 Figure S6  Artificial overexpression of *EPA1*. (A) Real-time quantitative PCR quantification of *EPA1* transcript levels. Whole-RNA extracts from log-phase *C. glabrata* cultures (VSY217, VSY220, and VSY221) in selective medium were used as the templates to synthesize cDNA. Transcript levels were measured using qPCR with specific primers and a TaqMan probe. Threshold cycles were normalized using *RDN5.8* quantification as the internal control. (B) Adherence to CHO-Lec2 epithelial cells. Mixed yeast suspensions of strains bearing the *PDR1^WT^* or the *PDR1^L280F^* alleles and an episomal plasmid for expression of *EPA1* under the control of the constitutive *S. cerevisiae* PGK1 promoter (or an empty plasmid control) in the DSY562 *C. glabrata* strain background (VSY217, VSY218, VSY220, and VSY221) were added to confluent CHO-Lec2 epithelial cell monolayers and incubated for 30 min at 37°C in a humid atmosphere with 5% CO_2_. The cultures were then washed four or five times to remove nonadherent yeasts, the epithelial cells were selectively lysed, and dilutions of the resulting yeast suspensions were plated on solid medium with or without fluconazole (to distinguish between azole-susceptible and azole-resistant yeast strains). Results are presented as CFU counts relative to the total inoculated CFU counts (set to 100%). Download Figure S6, TIF file, 0.8 MB. Copyright © 2016 Vale-Silva et al. 2016 Vale-Silva et al. This content is distributed under the terms of the Creative Commons Attribution 4.0 International license.

## DISCUSSION

### Effect of *PDR1* on *EPA1* expression.

We have previously established a link between *PDR1* hyperactivity and enhanced virulence in *C. glabrata* with mouse models of infection (12). A follow-up work showed that a decreased phagocytosis by macrophages and an increased adherence to host epithelial cells contribute to these phenotypes (25). Here, we report that regulation of these phenotypes by *PDR1* seems to be complex. *PDR1* hyperactivity *per se* does not guarantee differences in the interactions with host cells, which additionally depend on the *C. glabrata* genetic background and specific *PDR1* GOF mutations. Changes in phagocytosis by macrophages depend on the *C. glabrata* strain background, as phagocytosis of two of the four tested strains was not affected by replacement of the wild-type *PDR1* allele by *PDR1^L280F^*. Further work will be required to explain this observation. The interaction with epithelial cells, on the other hand, was affected in all strain backgrounds but depended on the specific GOF mutation. GOF mutations have profound and distinct effects on the *C. glabrata* transcriptome, and not all GOF mutations will lead to upregulation of *EPA1* (24). Until now, the molecular basis behind the transcriptome heterogeneity due to *PDR1* GOF mutations has not been elucidated. Among possible explanations, *PDR1* may recruit different cofactors or additional zinc cluster transcriptional regulators to form heterodimers, which may depend on the type of GOF mutation, thus shaping differences in target binding domains. On the other hand, *PDR1* may be subjected to selective degradation (for example, ubiquitylation) with different kinetics depending on the nature of GOF mutations, thus creating distinct transcriptional complexes and distinct transcriptional profiles. Transcriptional control by ubiquitylation is known for yeast transcription factors and especially when they are in an active state (31). Last, *PDR1* is known to interact with subunits of the Mediator complex, which is composed of a sophisticated architecture of proteins (32). Specific GOF mutations may interfere with the Mediator complex and alter transcription specificity and efficiency. Our data confirm that GPI-modified cell wall adhesin genes are part of the *PDR1* extensive regulon and show that *PDR1* modulates the interaction with epithelial cells through the transcriptional regulation of adhesins, among which *EPA1* was found to play a major role. In fact, increased adherence to epithelial cells in strains with hyperactive *PDR1* alleles strictly depends on *EPA1*. The question of whether *EPA1* regulation by *PDR1* is direct or indirect remains open. While deleting the putative PDRE on the promoter of *EPA1* resulted in a decrease in *EPA1* expression, chromatin immunoprecipitation (ChIP) experiments failed to detect a direct binding of Pdr1 to the *EPA1* promoter. It is possible that the subtelomeric location of *EPA1* makes Pdr1 difficult to access experimentally, due to the presence of other factors that are part of the subtelomeric silencing complex. These factors include Sir2 and other members (*HST1* to -*4*) of the bulky subtelomeric silencing protein complex (33). This might conceivably lead to a false-negative ChIP result, but more evidence is required to solve this question. *SIR2* downregulation is known to mediate derepression of *EPA1* (33). Genome-wide microarray data previously gathered by our laboratory did not involve *SIR2* downregulation in DSY565 (the clinical strain containing the *PDR1*^L280F^ allele) as a possible mediator of increased *EPA1* expression in azole-resistant isolates (24). As we mentioned above, *PDR1* may recruit the ubiquitylation machinery, which itself might be able to modify histones and therefore influence subtelomeric silencing and consequently *EPA1* expression. Ubiquitylation as an indirect regulatory mechanism was described in *S. cerevisiae* for ubiquitin-conjugating enzyme Rad6 and the histone H2B (34). This working model is under investigation in our laboratory.

### Challenges in addressing *EPA1* expression.

Adhesins have been notably absent from previous genome-wide studies of the Pdr1 regulon generally focused on analysis of azole drug resistance. The reason for this may be that adhesin genes are particularly difficult to analyze. Adhesin genes are characterized by the presence of megasatellites (tandemly repeated sequences with variable size), high intraspecies sequence variability, and high intergenic sequence similarity within the *EPA* family (35). These characteristics make these genes difficult to analyze in genome-wide transcriptional studies. In previous microarray experiments, *EPA* gene transcript signals may have been largely undetected and/or amalgamated with different transcript signals in the same family. A notable exception is the study by Caudle et al. (30), in which *EPA1* was upregulated by a specific *PDR1* GOF mutation (K274N). Interestingly, this work also highlighted a second *PDR1* GOF mutation (L946S), which did not result in *EPA1* upregulation and was thus in agreement with our observations on the different effects of GOF mutations on *EPA1* expression. Even in RNA sequencing experiments, adhesin genes may pose important challenges to mapping of short reads, which may exclude them from further analysis during quality control steps. Previous studies by the Cormack laboratory have mostly relied on radioactive probe-based S1 nuclease protection assay for specific and sensitive relative quantification of *EPA1* transcript levels (8, 28, 36, 37). Here, we have developed and used an alternative qPCR method. We have used strains harvested in mid-log phase following dilution of overnight saturated cultures and grown for two generations in rich medium. Testing *EPA1* expression under these conditions requires a strict attention to growth conditions to ensure reproducibility. This is especially relevant since *EPA1* expression changes rapidly during *in vitro* growth. It is transiently induced upon dilution in fresh growth medium under standard laboratory growth conditions and is then transcriptionally repressed by subtelomeric silencing (28).

### *C. glabrata* genetic backgrounds and *EPA1*-related phenotypes.

*EPA1* has long been considered the major adhesin of *C. glabrata*, enabling most of the capacity for adherence to epithelial cells, even though *C. glabrata* contains more than 20 *EPA*-like genes and other types of adhesin-like genes (6). We showed here that virulence-enhancing *PDR1* hyperactivity depends on *EPA1* to mediate increased adherence to epithelial cells. There are intraspecies differences in homogeneity of *EPA1* expression. Two *C. glabrata* groups of isolates have been identified, including the CBS138-like group of strains, in which *EPA1* expression is homogeneous in yeast cell populations, and BG2-like strains, in which expression varies between individual cells (38). These patterns also seem to be associated with differences in expression ratios between noninducing and inducing (NA limitation) conditions (38). DSY562 belongs to the CBS138-like group, as it shows homogeneous expression (data not shown). In this work, we also noticed intraspecies differences in basal levels of *EPA1* expression: transcript levels are highly variable between different *C. glabrata* strains (Fig. 5). Furthermore, there is yet another layer of complexity, since some *C. glabrata* strains are independent of *EPA1* for their high basal adherence to epithelial cells. While BG2 and DSY2235 depend almost exclusively on *EPA1* for adherence, DSY562 displays basal adherence levels independent of *EPA1*. In the DSY562 background, *EPA1* plays a role only in increased adherence. We have indications that the DSY562 genome contains a higher number of adhesin genes than BG2 or CBS138 (L. A. Vale-Silva and D. Sanglard, unpublished data). This feature may explain the relatively low dependence of this strain on *EPA1*. Interestingly, a recent study showed that *C. glabrata* isolates of clinical origins may harbor distinct patterns of cell wall adhesins contributing to the intrinsic adherence capacities of such strains (39). This reinforces the notion that *C. glabrata* isolates may differ between each other in their repertoire of adhesins by either differential expression and/or the presence of distinct genomic patterns.

### Relevance of *EPA1* in the UTI model.

We confirmed using the UTI model that *EPA1* is important for *in vivo* colonization of the bladder and kidneys. It is also crucial for *PDR1*-mediated increased colonization. This likely contributes to enhanced virulence. Previous data from the Cormack laboratory found no influence of an *epa1*Δ mutant in a vaginal and gastrointestinal infection model (7, 40). Later, the same laboratory found lower colonization of the mouse bladder by a mutant lacking three adhesion genes, including *EPA1*, *EPA6*, and *EPA7* (8, 40). We observed here that the single *EPA1* deletion is sufficient for decreasing *C. glabrata* fungal loads in the UTI mouse model. One reason for this discrepancy could be due to differences in genetic backgrounds of the investigated yeasts (BG2 versus DSY562/2235) and/or of the mice. It is likely that several other adhesins may play a role *in vivo* rather than *in vitro*. For example, deletion of *EPA1* in DSY2235 has a higher impact on adherence *in vitro* than on bladder/kidney colonization *in vivo*. On the other hand, overexpressing *EPA1* alone, even in the presence of *PDR1^WT^*, is enough to increase CFU to levels that may even reach saturation of the model.

In conclusion, we found that some adhesins, including *EPA1*, are regulated by *PDR1*. Elevated expression of *EPA1* is the major mechanism that leads to increased adherence to epithelial cells in strains bearing hyperactive *PDR1* alleles. *EPA1* has a clear impact *in vivo* and allows colonization of the bladder and kidneys in the UTI murine model. *EPA1* is likely to be an important contributor to increased virulence of azole-resistant *C. glabrata* strains with GOF *PDR1* alleles as we earlier published.

## MATERIALS AND METHODS

### *C*. *glabrata* strain culture and growth media.

All *C. glabrata* strains used in this study are listed in Table S1 in the supplemental material. Strains with the prefix SFY are from previously published collections (12, 24). All strains were stored in 20% glycerol stocks at −80°C and cultured in either YPD (1% yeast extract, 2% peptone, 2% d-glucose) rich medium or appropriate selective media at 30°C. For solid media, 2% agar was added. Selective medium for growth of transformed strains was YPD containing either 200 µg/ml of nourseothricin (clonNAT; Werner BioAgents, Germany) or 600 µg/ml of hygromycin B (PAA Laboratories, Austria). Additionally, YNB minimal medium (0.67% yeast nitrogen base plus 2% glucose) with appropriate amino acids and bases and without uracil was used to select uracil prototrophs. To obtain sensitive derivatives after recycling of the dominant selection marker cassettes, resistant strains were incubated for a minimum of 4 h in YCB-BSA medium (23.4 g/liter yeast carbon base and 4 g/liter bovine serum albumin; pH 4.0), and between 100 and 200 CFU was plated on YPD agar plates containing either 20 µg/ml of nourseothricin or 200 µg/ml of hygromycin B. YPD containing 30 µg/ml of fluconazole (Sigma) was used when required. *Escherichia coli* DH5α was used as a host for plasmid construction and propagation. *E. coli* DH5α was grown in Luria-Bertani broth or on Luria-Bertani agar plates, supplemented with 0.1 mg/ml of ampicillin (AppliChem GmbH, Germany) when required.

10.1128/mSphere.00065-15.7 Table S1  Strains used in this study Download Table S1, DOCX file, 0.1 MB. Copyright © 2016 Vale-Silva et al. 2016 Vale-Silva et al. This content is distributed under the terms of the Creative Commons Attribution 4.0 International license.

### Plasmids and *C*. *glabrata* strain constructions.

All yeast transformations were performed using an adapted lithium acetate (LiAc) procedure (41). Strains expressing fluorescent proteins were constructed using targeted gene disruption of *CgURA3*, followed by transformation with episomal plasmids complementing uracil auxotrophy as previously described (25). Inactivation of *URA3* in the DSY2235 strain background led to a detectable growth defect even after episomal *URA3* complementation. There was no difference between strains expressing different *PDR1* alleles and different fluorescent proteins (see Fig. S1 in the supplemental material). Other strain backgrounds had no detectable change after deletion of *URA3*.

*PDR1* deletion and replacement of *PDR1* alleles were performed using the recyclable dominant selection marker *SAT1* (42), encoding nourseothricin resistance (SAT^r^) as previously described (12).

*EPA1* deletion strains were constructed by targeted gene disruption of *EPA1* using recyclable dominant selection markers. A recyclable marker based on the *hph* gene from *Klebsiella pneumoniae*, encoding hygromycin B resistance (Hyg^r^) (8), was designed to use with SAT^r^ strains. To construct this plasmid, the *hph* expression cassette (FLP recombination target [FRT]-*ScPGK1p-hph*-3' untranslated region [UTR] *ScHIS3*-FRT) was amplified by PCR from plasmid pAP599 (8), a gift from Brendan Cormack (Johns Hopkins University), using primers HygR-BlpI (5′-GGCCGCTTAGCGAGAAAGAAATTACCGTC-3′) and HygR-NotI (5′-GTCGGCGGCCGCGATAAGCTTGAAGTTC-3′). The amplicon was inserted into BlpI/NotI-digested pSFS1A, a gift from Joachim Morschhäuser (Universität Würzburg), in order to replace the *SAT1*-expressing section of the *SAT1* flipper, thus generating pVS31. To construct the *EPA1* disruption cassettes, the complete *EPA1* open reading frame (ORF) flanked by 500 bp was amplified by PCR from genomic DNA of DSY562 using primers EPA1-KpnI (5′-TCAAGGTACCTACTAAGGTTCCATGGCTG-3′) and EPA1-SacII-2 (5′-CCTACCGCGGTTTCTTTTCACCTGAAAGATTAC-3′). The resulting PCR product was inserted into pBluescript II KS(+) to generate pVS33. This plasmid was amplified by PCR using primers EPA1-XhoI (5′-AAATCTCGAGAATGAAGAAAAAGCTTTGTGAAGGC-3′) and EPA1-NotI (5′-CATAGCGGCCGCAAACCAGGAAATATAATAACTTCC-3′). The resulting PCR product was digested by XhoI and NotI and ligated to XhoI/NotI fragments from either pSFS1A or pVS31. The resulting plasmids pVS34 (*SAT1* flipper-based *EPA1* deletion cassette) and pVS37 (*hph*-based *EPA1* deletion cassette) were digested by SacII and KpnI or SacI and KpnI, respectively, and used to transform *C. glabrata* strains.

To construct the *EPA1* reversion cassette, the *EPA1* ORF flanked by 500 bp and 1 kb was amplified by PCR from DSY562 genomic DNA using primers EPA1-KpnI (5′-TCAAGGTACCTACTAAGGTTCCATGGCTG-3′) and EPA1-SacII-3 (5′-TGCTCCGCGGCCATGAGATTATCTTCTTGAAAG-3′) and inserted into pBluescript II KS(+) to generate pVS42. This plasmid was amplified by PCR using primers 3′ UTR-EPA1-NotI (5′-AAAAGCGGCCGCGTTAGGCTTATTAGAACCAG-3′) and 3′ UTR-EPA1-XhoI (5′-CTAACTCGAGTTCTTTTCACCTGAAAGATTAC-3′), digested by XhoI and NotI, and ligated to the XhoI/NotI fragment from pSFS1A, thus generating pVS43. The resulting plasmid was digested by SacII and KpnI and used to transform *C. glabrata* strains. Recycling of the *SAT1* flipper reconstitutes the native 3′-flanking region of the gene, leaving only one 34-bp flippase recombination target (FRT) sequence at about 600 bp downstream of the stop codon.

To construct *EPA1* overexpression strains, the complete *EPA1* ORF was amplified by PCR from DSY562 genomic DNA, using primers EPA1-XbaI (5′-CCAATCTAGAACAATGATTTTAAATCCAGCTC-3′) and EPA1-XhoI-2 (5′-AATGCTCGAGTATGGAAGTTATTATATTTCCTGG-3′), digested by XbaI and XhoI, and ligated to a XbaI/XhoI fragment from vector pGRB2.3 (43). This generated pVS39, which is episomal (CgCEN/ARS) and contains *URA3*. It contains the *EPA1* overexpression cassette (*ScPGK1p-EPA1-ScHIS3*) replacing the *yEGFP* gene from the parent pGRB2.3. The same vector containing only the promoter and the terminator, pVS20 (25), was used as the parent control plasmid. The nondigested plasmids were used to transform *ura3*Δ *C. glabrata* strains. Transformants were selected on YNB lacking uracil.

To delete the putative PDRE on positions −571 to −564 relative to the *EPA1* start codon (a possible Pdr1 binding site), a 1-kb *EPA1* 5′-flanking sequence was amplified by PCR using primers EPA1-KpnI-2 (5′-GCAGGGTACCAAAAAGAACATC-3′) and EPA1-NcoI (5′-GTCACCATGGAATAGAGTGGATCTGGTACTTTG-3′). The PCR product was digested by KpnI and NcoI and ligated to a KpnI/NcoI fragment from pVS43, thus generating pVS47. This makes use of a naturally occurring NcoI restriction site 35 bp downstream of the putative PDRE and generates a plasmid carrying a deletion of 72 bp (between positions −600 and −527) centered on the putative PDRE on the 5′ UTR of *EPA1*. pVS47 was digested by SacII and KpnI and used to transform *epa1*Δ *C. glabrata* strains.

All constructed plasmids were confirmed by DNA sequencing. Constructed strains were confirmed by sequencing or PCR. *EPA1* deletions and reversions were confirmed by Southern blotting (data not shown).

### Epithelial cell and macrophage cultures.

Mammalian cell lines were cultured at 37°C in a humid atmosphere with 5% CO_2_. Chinese hamster ovary cell lines Lec2 (CHO-Lec2; ATCC CRL1736) and AA8 (CHO-AA8; CRL-1859) were cultured in complete minimum essential medium α (MEM-α): high-glucose minimum essential medium α (Life Technologies, Zug, Switzerland) with l-glutamine and supplemented with 100 U/ml penicillin, 100 µg/ml streptomycin (Life Technologies), and 10% fetal bovine serum (FBS) (Life Technologies). Human cervix adenocarcinoma cells (CHO-HeLa; ATCC CCL-2), human colorectal adenocarcinoma cells (Caco-2; ATCC HTB-37), and mouse rectum polyploidy carcinoma cells (CMT-93; ATCC CCL-223) were cultured in complete Dulbecco’s modified Eagle’s medium (DMEM): high-glucose Dulbecco’s modified Eagle’s medium with GlutaMAX (Life Technologies), supplemented with 100 U/ml penicillin and 100 µg/ml streptomycin (Life Technologies) and 10% fetal bovine serum (FBS; Life Technologies). Abelson murine leukemia virus-induced tumor macrophage-like cells (RAW 264.7; ATCC TIB-71) were also routinely cultured in complete DMEM.

### Phagocytosis assays.

Competition phagocytosis assays were performed as previously described (25). Briefly, harvested bone marrow-derived macrophages (BMDMs) were suspended in complete Iscove modified Dulbecco medium (IMDM) at a density of 3.0 × 10^5^ cells/ml. RAW 264.7 macrophage-like cells were suspended in complete DMEM at a density of 1.0 × 10^5^ cells/ml in complete IMDM. Macrophage suspensions were transferred to 24-well plates in a volume of 1 ml per well on top of round cover slides and incubated overnight at 37°C in a humid atmosphere with 5% CO_2_. Macrophages were then infected with 1:1 mixtures of 3.0 × 10^5^ yeast cells of each of the two *C. glabrata* strains in 100 µl of phosphate-buffered saline solution (PBS). The plates were centrifuged for 1 min at 200 × *g* and incubated for 30 min at 37°C in the presence of 5% CO_2_. Cocultures were washed to remove non-macrophage-associated yeasts, and the round cover slides with adherent cells were stained with 100 µg/ml of calcofluor white for 10 min. The cover slides were mounted onto microscopy slides and observed using a Zeiss Axioplan 2 epifluorescence microscope. Images were recorded using a Visitron Systems HistoScope camera and VisiView imaging software (Visitron Systems, Puchheim, Germany).

### Yeast adherence to epithelial cells.

Adherence to epithelial cells was tested using our protocol (25) adapted from a previously published assay (7). Briefly, log-phase epithelial cells were seeded in 24-well plates at a density of 1.0 × 10^5^ cells/well in 1 ml of culture medium and allowed to grow to full confluence at 37°C in a humid atmosphere with 5% CO_2_, typically for 48 to 72 h. To prepare *C. glabrata* suspensions for infection, overnight cultures of test strains were diluted in fresh medium and grown for a minimum of two generations to mid-log phase. For nicotinic acid limitation experiments, yeast strains were grown either in complete YNB minimal medium (0.67% yeast nitrogen base plus 2% glucose) with appropriate amino acids and bases or in the same medium containing only 5% of the normal nicotinic acid concentration (nicotinic acid limitation). Log-phase cultures were washed and resuspended in PBS. Epithelial cell monolayers were infected with 1:1 mixed yeast suspensions containing 3.0 × 10^5^ yeast cells, and the plates were centrifuged at 200 × *g* for 1 min. Cocultures were incubated at 37°C in a humid atmosphere with 5% CO_2_ for 30 min, and nonadherent yeasts were removed by washing. Adherent yeasts were recovered by lysis of the epithelial cells in 0.1% Triton X-100 and plated onto YPD agar plates for quantification of CFU. YPD agar alone and YPD agar plates containing 30 µg/ml of fluconazole were used to distinguish between azole-susceptible and azole-resistant yeast strains.

### qPCR.

Total RNA was extracted from log-phase cultures in 5 ml of YPD broth by mechanical disruption of the cells with glass beads as previously described (44). Total RNA extracts were treated with DNase using the DNA-free kit (Ambion-Life Technologies, Zug, Switzerland), and 1 µg of RNA was used as a template to synthesize cDNA using the Transcriptor high-fidelity cDNA synthesis kit (Roche Diagnostics, Rotkreuz, Switzerland). For relative quantification of the target genes, real-time quantitative PCRs (qPCRs) were performed using sets of primers and TaqMan probes with the iTaq Supermix with ROX (Bio-Rad Laboratories AG, Cressier, Switzerland) in a StepOnePlus real-time PCR system (Applied Biosystems-Life Technologies, Zug, Switzerland). The specific primers and probe used for the target gene *EPA1* were EPA1a (5′-ACCGCAAGAAAATCCTCCTCC-3′), EPA1b (5′-TGGTGCTGATGATATTGATTTGTTG-3′), and EPA1pr (6-carboxyfluorescein [FAM]–5′-TGGCCTCCATTCATACCCCACTTCCA-3′–6-carboxytetramethylrhodamine [TAMRA]). The specific primers and probe used for the target gene *EPA7* were EPA7a (5′-TGATTTACGGAAGAATGGTTCG-3′), EPA7b (5′-TTACCGGTAACACCATCAACT-3′), and EPA7pr (6-FAM–5′-TGGGATCTAAATATGCGGCATCCCAACA-3′–TAMRA). The specific primers and probe used for the target gene *EPA12* were EPA12a (5′-AAGGGTTTGTCAATGGAACTG-3′), EPA12b (5′-CACCCTTGGAAAATTCGGATC-3′), and EPA12pr (6-FAM–5′-TCGGAAGAAAGGTTCTCACCCATGCT-3′–TAMRA). The specific primers and probe used for the target gene *PWP4* were PWP4a (5′-GAGTAGATCTAGAACTGCGGG-3′), PWP4b (5′-AGTGATCAACTGGGAACTACC-3′), and PWP4pr (6-FAM–5′-ACCCAGCCCTGCAGTGAGTACTCT-3′–TAMRA). The internal control gene was *RDN5.8*, using previously published oligonucleotides (45): RDN5.8-F (5′-CTTGGTTCTCGCATCGATGA-3′), RDN5.8-R (5′-GGCGCAATGTGCGTTCA-3′), and RDN5.8-Pr (6-FAM–5′-ACGCAGCGAAATGCGATACGTAATGTG-3′–TAMRA). At least three biological replicates were included, and each reaction was run in duplicate. Changes (*n*-fold) in gene transcription levels relative to an included reference *C. glabrata* strain were determined from *RDN5*.*8-1*-normalized threshold cycle (*C_T_*) values. A 2-fold increase in transcript level was arbitrarily considered significant.

### UTI model.

Urinary tract infection (UTI) experiments were performed according to a previously described model (46). Briefly, for tissue burden experiments, each *C. glabrata* strain was grown in 10 ml of YPD broth under agitation for 18 h at 37°C. After growth, cells were centrifuged, washed and resuspended in 10 ml of sterile PBS, and then adjusted to reach a concentration of 5 × 10^8^ cells/ml. For each strain, a group of 10 isoflurane-anesthetized female BALB/c mice were infected via intraurethral catheterization (polyethylene catheter, about 4 cm long; outer diameter, 0.61 mm; Becton, Dickinson, Sparks, MD) using 100 µl of the corresponding *C. glabrata* cell suspension for each animal. Mice were sacrificed 7 days after the transurethral challenge, and for each animal, bladders and kidney pairs were harvested, weighed, and homogenized in 1 and 5 ml of sterile saline, respectively. *C. glabrata* inocula and burdens were enumerated by performing serial dilutions and counting CFU on YPD agar. The *C. glabrata* detection limits were 50 and 10 CFU/ml for kidneys and bladder homogenates, respectively. CFU counts were analyzed by unpaired *t* tests, and a *P* value of less than 0.05 was considered significant.

### Nucleotide sequence accession numbers.

The *EPA1* gene sequences of *C. glabrata* strains DSY562 and DSY2235 determined in the present study have been deposited in GenBank under accession numbers KR296804 and KR296805, respectively.
