# Supplementary figures and images for: Upregulation of the Adhesin Gene EPA1 Mediated by PDR1 in Candida glabrata Leads to Enhanced Host Colonization
Source: mSphere. 2016 Mar 2;1(2):e00065-15. doi: 10.1128/mSphere.00065-15 (PMC4863579; doi:10.1128/mSphere.00065-15)

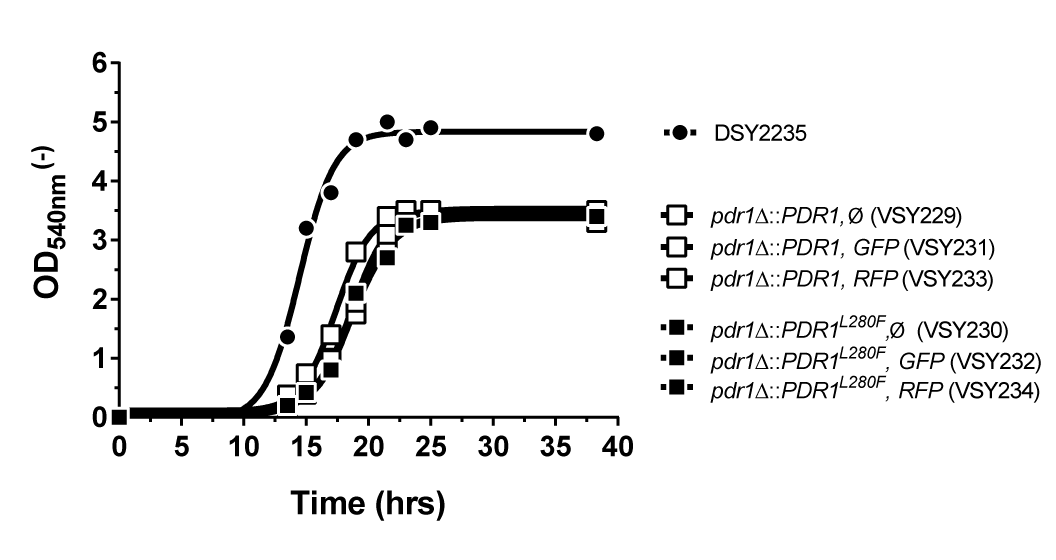

Supplement: Figure S1 [file sph001162034sf1.tif]

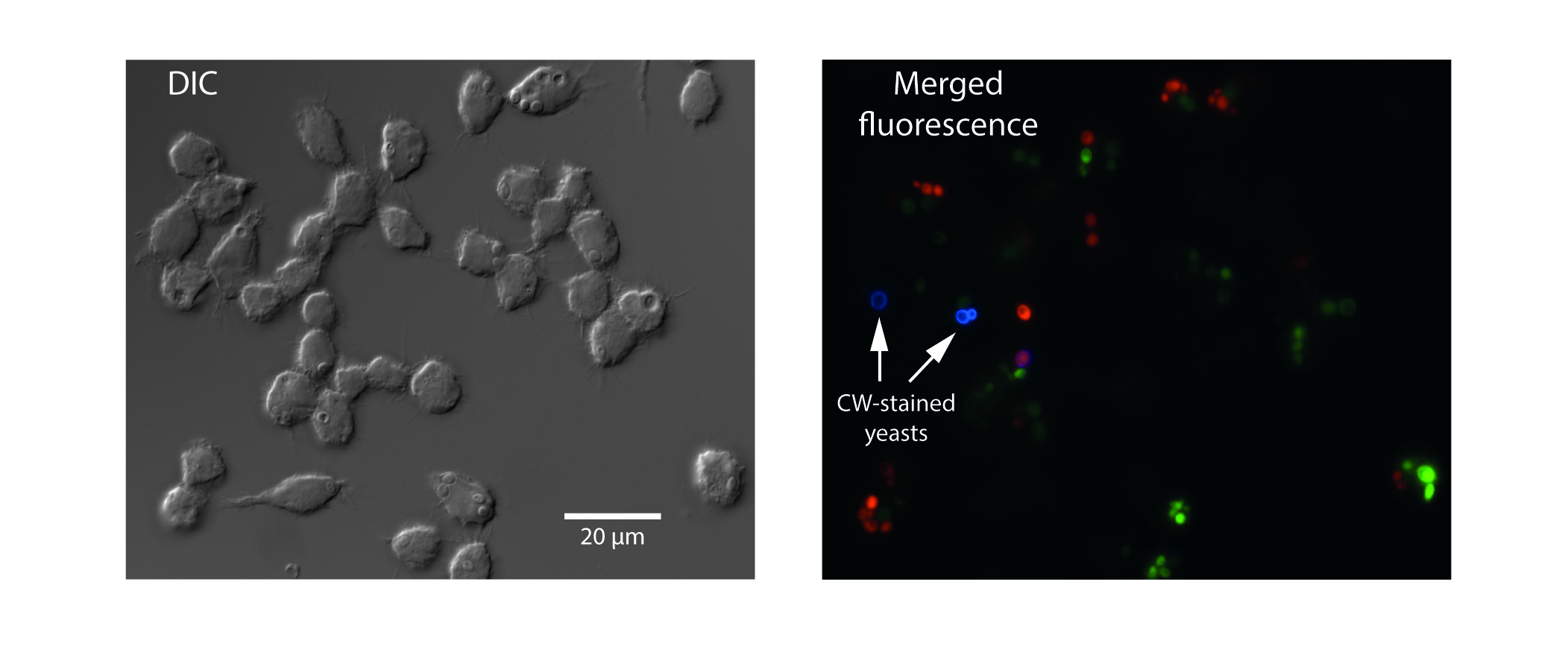

Supplement: Figure S2 [file sph001162034sf2.tif]

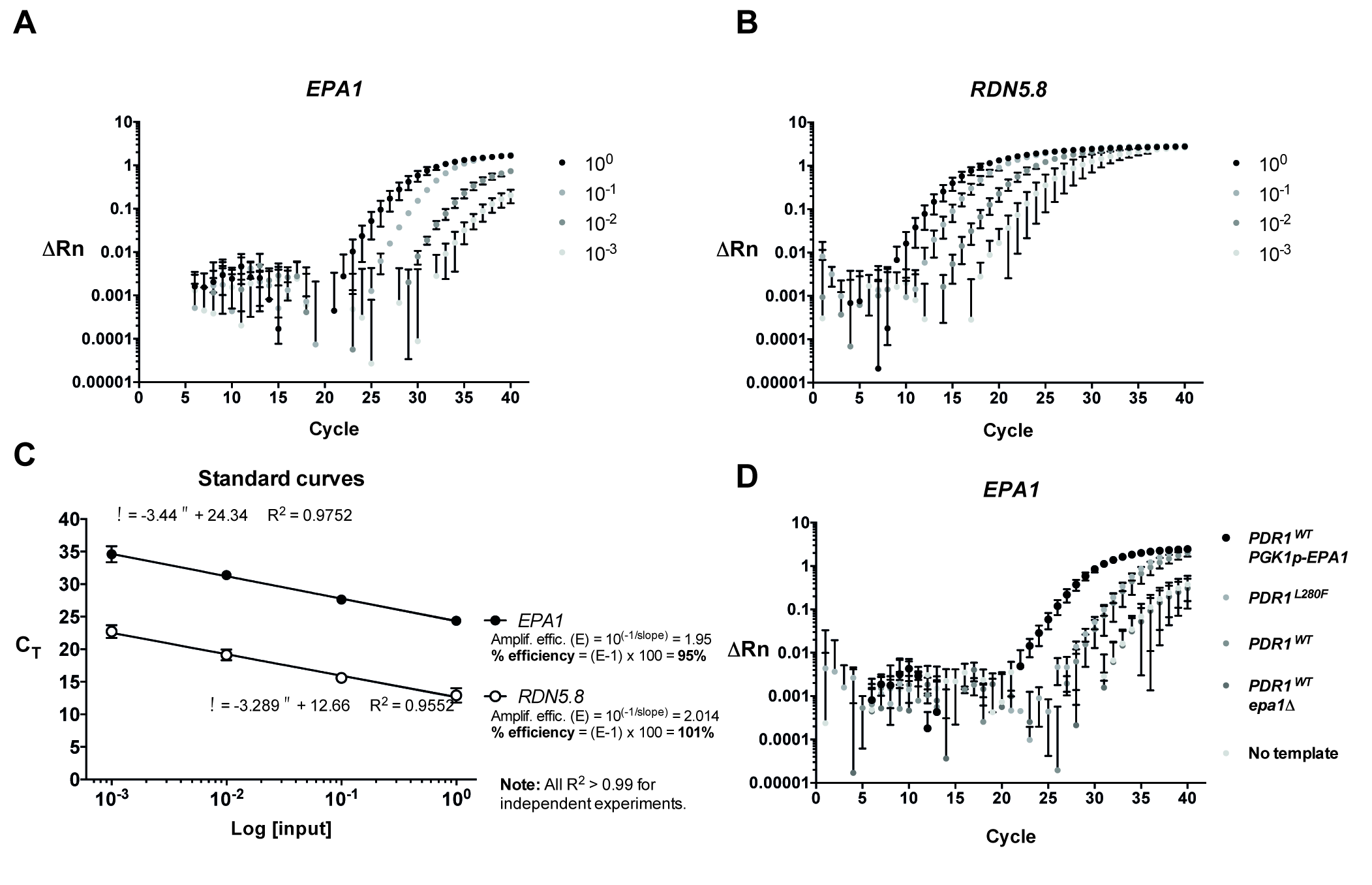

Supplement: Figure S3 [file sph001162034sf3.tif]

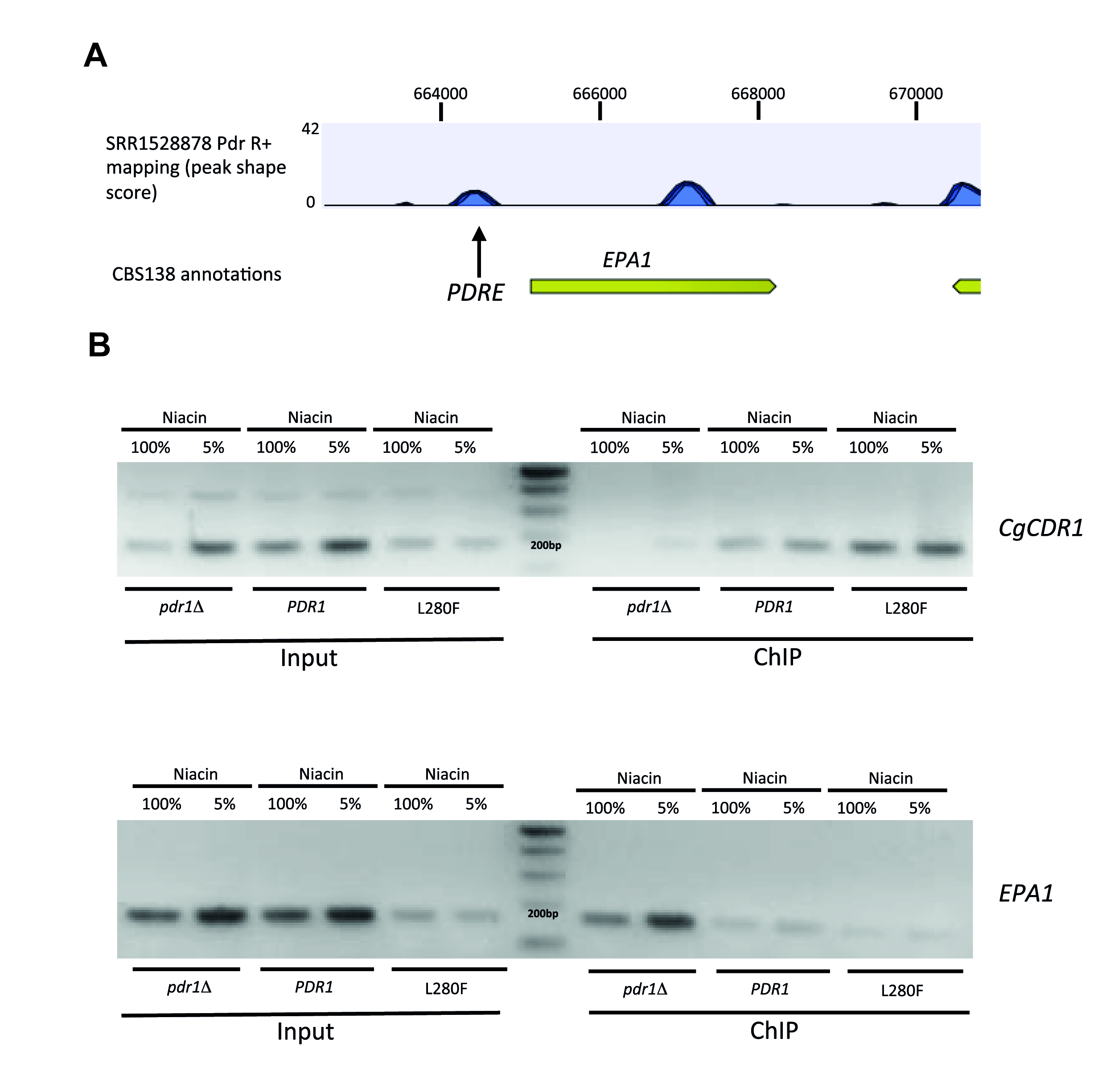

Supplement: Figure S4 [file sph001162034sf4.tif]

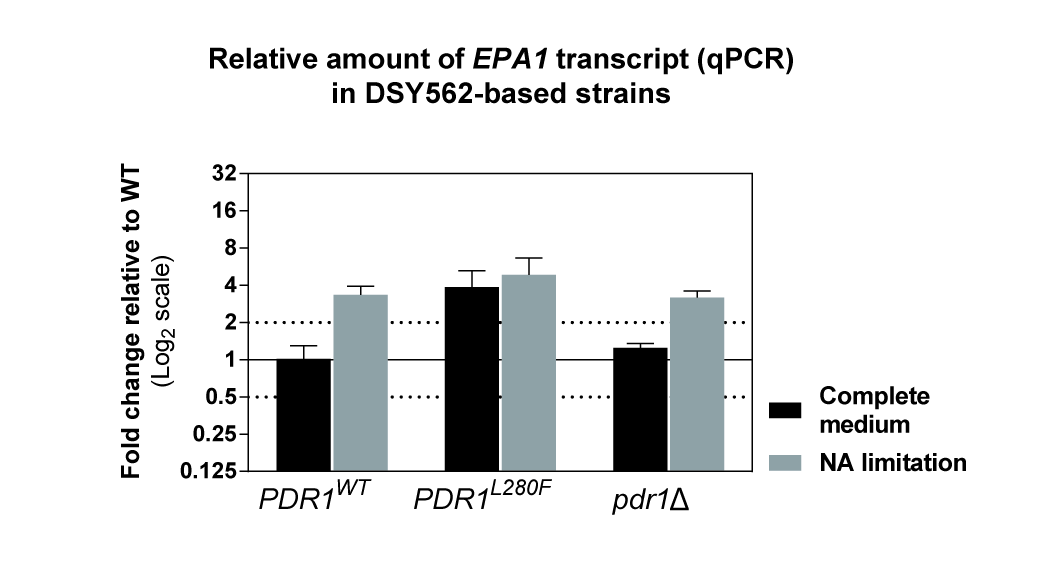

Supplement: Figure S5 [file sph001162034sf5.tif]

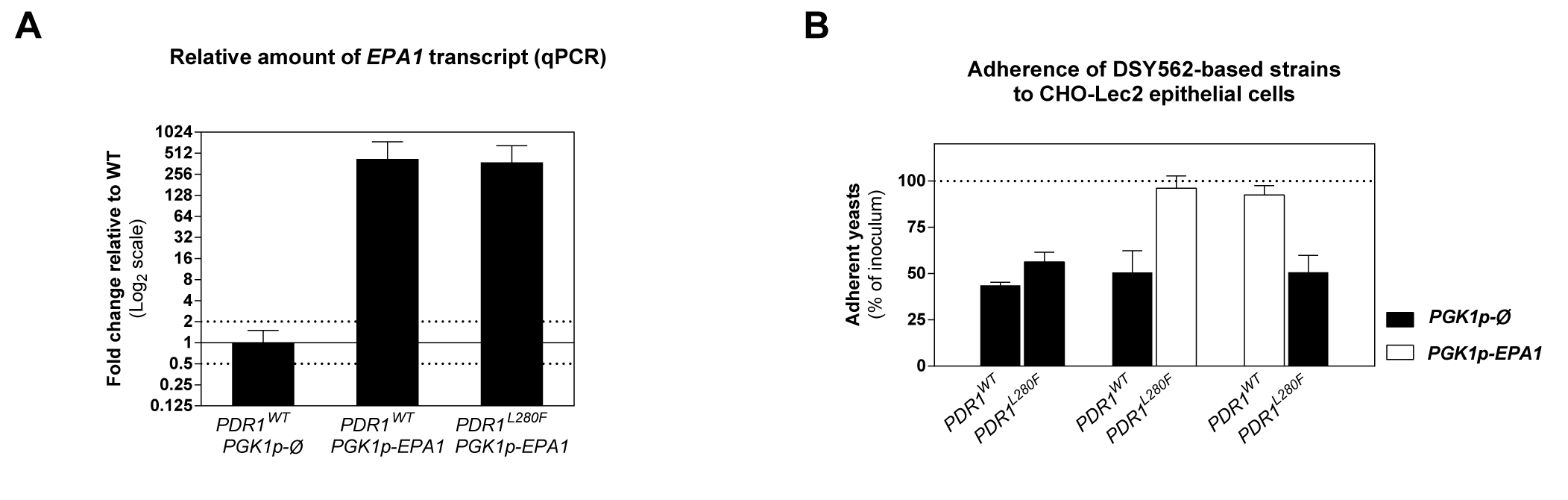

Supplement: Figure S6 [file sph001162034sf6.tif]
