# Supplementary material for: Upregulation of the Adhesin Gene EPA1 Mediated by PDR1 in Candida glabrata Leads to Enhanced Host Colonization
Source: mSphere. 2016 Mar 2;1(2):e00065-15. doi: 10.1128/mSphere.00065-15 (PMC4863579; doi:10.1128/mSphere.00065-15)
Supplement: Table S1 [file sph001162034st7.docx]

Supplementary Table S1 : Strains used in this study

| **Strain** | **Parent strain** | **Plasmid used** | **Genotype** | **Reference** |
| --- | --- | --- | --- | --- |
| DSY562 | - | - | Azole-susceptible clinical isolate | (1) |
| DSY565 | Related to DSY562 | - | Azole-resistant clinical isolate | (1) |
| SFY93 | SFY92 | pSF2 | DSY562 *pdr1::FRT* | (1) |
| SFY114 | SFY93 | pSF66 | DSY562 *pdr1::PDR1-SAT1* | (1) |
| SFY115 | SFY93 | pSF67 | DSY562 *pdr1::PDR1^L280F^-SAT1* | (1) |
| DSY2235 | - | - | Azole-susceptible clinical isolate | (1) |
| BG2 | - | - | Clinical isolate | (2) |
| BG14 | BG2 | pBC39.1 | BG2 *ura3::Tn903* | (2) |
| CBS138/ ATCC2001 | - | - | Clinical isolate | (3) |
| VSY21 | DSY562 | pVS13 | DSY562 *ura3::hph* | (4) |
| VSY22 | DSY565 | pVS13 | DSY565 *ura3::hph* | (4) |
| VSY43 | SFY114 | pVS13 | DSY562 *pdr1::PDR1-SAT1*, *ura3::hph* | (4) |
| VSY44 | SFY115 | pVS13 | DSY562 *pdr1::PDR1^L280F^-SAT1*, *ura3::hph* | (4) |
| VSY101 | VSY43 | pVS20 | DSY562 *pdr1::PDR1-SAT1 ura3::hph*,  *ScPGK1p-URA3-CEN-ARS* | (4) |
| VSY102 | VSY44 | pVS20 | DSY562 *pdr1::PDR1^L280F^-SAT1*, *ura3::hph*, *ScPGK1p-URA3-CEN-ARS* | (4) |
| VSY103 | VSY43 | pGRB2.3 | DSY562 *pdr1::PDR1-SAT1*, *ura3::hph*,  *ScPGK1p-yEGFP-URA3-CEN-ARS* | (4) |
| VSY104 | VSY44 | pGRB2.3 | DSY562 *pdr1::PDR1^L280F^-SAT1*, *ura3::hph*, *ScPGK1p-yEGFP-URA3-CEN-ARS* | (4) |
| VSY105 | VSY43 | pVS19 | DSY562 *pdr1::PDR1-SAT1*, *ura3::hph*,  *ScPGK1p-yEmRFP-URA3-CEN-ARS* | (4) |
| VSY106 | VSY44 | pVS19 | DSY562 *pdr1::PDR1^L280F^-SAT1*, *ura3::hph*, *ScPGK1p-yEmRFP-URA3-CEN-ARS* | (4) |
| VSY119 | SFY93 | pVS13 | DSY562 *pdr1::FRT*, *ura3::hph* | (4) |
| VSY120 | SFY95 | pVS13 | DSY565 *pdr1::FRT*, *ura3::hph* | (4) |
| VSY132 | SFY101 | pVS13 | DSY562 *pdr1::PDR1^R376W^-SAT1*, *ura3::hph* | (4) |
| VSY133 | SFY105 | pVS13 | DSY562 *pdr1::PDR1^T588A^-SAT1*, *ura3::hph* | (4) |
| VSY134 | VSY132 | pVS20 | DSY562 *pdr1::PDR1^R376W^-SAT1*, *ura3::hph*, *ScPGK1p-URA3-CEN-ARS* | (4) |
| VSY135 | VSY133 | pVS20 | DSY562 *pdr1::PDR1^T588A^-SAT1*, *ura3::hph*, *ScPGK1p-URA3-CEN-ARS* | (4) |
| VSY136 | VSY132 | pGRB2.3 | DSY562 *pdr1::PDR1^R376W^-SAT1*, *ura3::hph*, *ScPGK1p-yEGFP-URA3-CEN-ARS* | (4) |
| VSY137 | VSY133 | pGRB2.3 | DSY562 *pdr1::PDR1^T588A^-SAT1*, *ura3::hph*, *ScPGK1p-yEGFP-URA3-CEN-ARS* | (4) |
| VSY138 | VSY132 | pVS19 | DSY562 *pdr1::PDR1^R376W^-SAT1*, *ura3::hph*, *ScPGK1p-yEmRFP-URA3-CEN-ARS* | (4) |
| VSY139 | VSY133 | pVS19 | DSY562 *pdr1::PDR1^T588A^-SAT1*, *ura3::hph*, *ScPGK1p-yEmRFP-URA3-CEN-ARS* | (4) |
| VSY140 | BG2 | pSF2 | BG2 *pdr1::SAT1-FLIP* | This study |
| VSY141 | BG14 | pSF2 | BG2 *ura3::Tn903, pdr1::SAT1-FLIP* | This study |
| VSY142 | VSY140 | - | BG2 *pdr1::FRT* | This study |
| VSY143 | VSY141 | - | BG2 *ura3::Tn903, pdr1::FRT* | This study |
| VSY144 | VSY143 | pSF66 | BG2 *ura3::Tn903, pdr1::PDR1-SAT1* | This study |
| VSY145 | VSY143 | pSF67 | BG2 *ura3::Tn903, pdr1::PDR1^L280F^-SAT1* | This study |
| VSY146 | VSY143 | pSF53 | BG2 *ura3::Tn903, pdr1::PDR1^R376W^-SAT1* | This study |
| VSY147 | VSY143 | pSF57 | BG2 *ura3::Tn903, pdr1::PDR1^T588A^-SAT1* | This study |
| VSY148 | VSY143 | pVS20 | BG2 *ura3::Tn903, pdr1::FRT, ScPGK1p-URA3-CEN-ARS* | This study |
| VSY149 | VSY144 | pVS20 | BG2 *ura3::Tn903, pdr1::PDR1-SAT1, ScPGK1p-URA3-CEN-ARS* | This study |
| VSY150 | VSY145 | pVS20 | BG2 *ura3::Tn903, pdr1*:: *PDR1^L280F^-SAT1, ScPGK1p-URA3-CEN-ARS* | This study |
| VSY151 | VSY146 | pVS20 | BG2 *ura3::Tn903, pdr1::PDR1^R376W^-SAT1, ScPGK1p-URA3-CEN-ARS* | This study |
| VSY152 | VSY147 | pVS20 | BG2 *ura3::Tn903, pdr1::PDR1^T588A^-SAT1, ScPGK1p-URA3-CEN-ARS* | This study |
| VSY153 | VSY143 | pGRB2.3 | BG2 *ura3::Tn903, pdr1::FRT, ScPGK1p- yEGFP-URA3-CEN-ARS* | This study |
| VSY154 | VSY144 | pGRB2.3 | BG2 *ura3::Tn903, pdr1::PDR1-SAT1, ScPGK1p- yEGFP-URA3-CEN-ARS* | This study |
| VSY155 | VSY145 | pGRB2.3 | BG2 *ura3::Tn903, pdr1::PDR1^L280F^-SAT1, ScPGK1p- yEGFP-URA3-CEN-ARS* | This study |
| VSY156 | VSY146 | pGRB2.3 | BG2 *ura3::Tn903, pdr1::PDR1^R376W^-SAT1, ScPGK1p- yEGFP-URA3-CEN-ARS* | This study |
| VSY157 | VSY147 | pGRB2.3 | BG2 *ura3::Tn903, pdr1::PDR1^T588A^-SAT1, ScPGK1p- yEGFP-URA3-CEN-ARS* | This study |
| VSY158 | VSY143 | pVS19 | BG2 *ura3::Tn903, pdr1::FRT, ScPGK1p-yEmRFP-URA3-CEN-ARS* | This study |
| VSY159 | VSY144 | pVS19 | BG2 *ura3::Tn903, pdr1::PDR1-SAT1, ScPGK1p- yEmRFP-URA3-CEN-ARS* | This study |
| VSY160 | VSY145 | pVS19 | BG2 *ura3::Tn903, pdr1::PDR1^L280F^-SAT1, ScPGK1p-yEmRFP-URA3-CEN-ARS* | This study |
| VSY161 | VSY146 | pVS19 | BG2 *ura3::Tn903, pdr1::PDR1^R376W^-SAT1, ScPGK1p-yEmRFP-URA3-CEN-ARS* | This study |
| VSY162 | VSY147 | pVS19 | BG2 *ura3::Tn903, pdr1::PDR1^T588A^-SAT1, ScPGK1p-yEmRFP-URA3-CEN-ARS* | This study |
| VSY164 | VSY22 | pVS34 | DSY565 *ura3::hph, epa1::SAT1-FLIP* | This study |
| VSY166 | VSY164 | - | DSY565 *ura3*::*hph, epa1::FRT* | This study |
| VSY182 | CBS138 | pVS13 | CBS138 *ura3::hph* | This study |
| VSY183 | VSY182 | pSF2 | CBS138 *ura3*::*hph, pdr1::SAT1-FLIP* | This study |
| VSY184 | VSY183 | - | CBS138 *ura3::FRT, pdr1::FRT* | This study |
| VSY186 | DSY2235 | pVS13 | DSY2235 *ura3*::*hph* | This study |
| VSY188 | VSY186 | pSF2 | DSY2235 *ura3::hph, pdr1::SAT1-FLIP* | This study |
| VSY190 | VSY188 | - | DSY2235 *ura3::FRT, pdr1::FRT* | This study |
| VSY213 | VSY190 | pSF66 | DSY2235 *ura3::FRT, pdr1::PDR1-SAT1* | This study |
| VSY214 | VSY190 | pSF67 | DSY2235 *ura3::FRT, pdr1::PDR1^L280F^-SAT1* | This study |
| VSY215 | VSY184 | pSF66 | CBS138 *ura3::FRT, pdr1::PDR1-SAT1* | This study |
| VSY216 | VSY184 | pSF67 | CBS138 *ura3::FRT, pdr1::PDR1^L280F^-SAT1* | This study |
| VSY217 | VSY43 | pVS20 | DSY562 *pdr1::PDR1-SAT1*, *ura3*::*hph, ScPGK1p-URA3-CEN-ARS* | This study |
| VSY218 | VSY44 | pVS20 | DSY562 *pdr1::PDR1^L280F^-SAT1*, *ura3::hph, ScPGK1p-URA3-CEN-ARS* | This study |
| VSY220 | VSY43 | pVS39 | DSY562 *pdr1::PDR1-SAT1*, *ura3*::*hph, ScPGK1p- EPA1-URA3-CEN-ARS* | This study |
| VSY221 | VSY44 | pVS39 | DSY562 *pdr1::PDR1^L280F^-SAT1*, *ura3::hph, ScPGK1p-EPA1-URA3-CEN-ARS* | This study |
| VSY229 | VSY213 | pVS20 | DSY2235 *ura3::FRT, pdr1::PDR1-SAT1, ScPGK1p-URA3-CEN-ARS* | This study |
| VSY230 | VSY214 | pVS20 | DSY2235 *ura3::FRT, pdr1::PDR1^L280F^-SAT1, ScPGK1p-URA3-CEN-ARS* | This study |
| VSY231 | VSY213 | pGRB2.3 | DSY2235 *ura3::FRT, pdr1::PDR1-SAT1, ScPGK1p- yEGFP-URA3-CEN-ARS* | This study |
| VSY232 | VSY214 | pGRB2.3 | DSY2235 *ura3::FRT, pdr1::PDR1^L280F^-SAT1, ScPGK1p-yEGFP-URA3-CEN-ARS* | This study |
| VSY233 | VSY213 | pVS19 | DSY2235 *ura3::FRT, pdr1::PDR1-SAT1, ScPGK1p- yEmRFP-URA3-CEN-ARS* | This study |
| VSY234 | VSY214 | pVS19 | DSY2235 *ura3::FRT, pdr1::PDR1^L280F^-SAT1, ScPGK1p-yEmRFP-URA3-CEN-ARS* | This study |
| VSY236 | VSY215 | pVS20 | CBS138 *ura3::FRT, pdr1::PDR1-SAT1, ScPGK1p-URA3-CEN-ARS* | This study |
| VSY237 | VSY216 | pVS20 | CBS138 *ura3::FRT, pdr1::PDR1^L280F^-SAT1, ScPGK1p-URA3-CEN-ARS* | This study |
| VSY239 | VSY215 | pGRB2.3 | CBS138 *ura3::FRT, pdr1::PDR1-SAT1, ScPGK1p- yEGFP-URA3-CEN-ARS* | This study |
| VSY240 | VSY216 | pGRB2.3 | CBS138 *ura3::FRT, pdr1::PDR1^L280F^-SAT1, ScPGK1p-yEGFP-URA3-CEN-ARS* | This study |
| VSY242 | VSY215 | pVS19 | CBS138 *ura3::FRT, pdr1::PDR1-SAT1, ScPGK1p- yEmRFP-URA3-CEN-ARS* | This study |
| VSY243 | VSY216 | pVS19 | CBS138 *ura3::FRT, pdr1::PDR1^L280F^-SAT1, ScPGK1p-yEmRFP-URA3-CEN-ARS* | This study |
| VSY244 | VSY166 | pVS20 | DSY565 *ura3*::*hph, epa1::FRT, ScPGK1p-URA3-CEN-ARS* | This study |
| VSY245 | VSY166 | pGRB2.3 | DSY565 *ura3*::*hph, epa1::FRT, ScPGK1p-yEGFP-URA3-CEN-ARS* | This study |
| VSY246 | VSY166 | pVS19 | DSY565 *ura3*::*hph, epa1::FRT, ScPGK1p-yEmRFP-URA3-CEN-ARS* | This study |
| VSY250 | VSY184 | pSF53 | CBS138 *ura3::FRT, pdr1::PDR1^R376W^-SAT1* | This study |
| VSY251 | VSY184 | pSF57 | CBS138 *ura3::FRT, pdr1::PDR1^T588A^-SAT1* | This study |
| VSY254 | VSY250 | pVS20 | CBS138 *ura3::FRT, pdr1::PDR1^R376W^-SAT1, ScPGK1p-URA3-CEN-ARS* | This study |
| VSY255 | VSY251 | pVS20 | CBS138 *ura3::FRT, pdr1::PDR1^T588A^-SAT1, ScPGK1p-URA3-CEN-ARS* | This study |
| VSY258 | VSY250 | pGRB2.3 | CBS138 *ura3::FRT, pdr1::PDR1^R376W^-SAT1, ScPGK1p-yEGFP-URA3-CEN-ARS* | This study |
| VSY259 | VSY251 | pGRB2.3 | CBS138 *ura3::FRT, pdr1::PDR1^T588A^-SAT1, ScPGK1p-yEGFP-URA3-CEN-ARS* | This study |
| VSY262 | VSY250 | pVS19 | CBS138 *ura3::FRT, pdr1::PDR1^R376W^-SAT1, ScPGK1p-yEmRFP-URA3-CEN-ARS* | This study |
| VSY263 | VSY251 | pVS19 | CBS138 *ura3::FRT, pdr1::PDR1^T588A^-SAT1, ScPGK1p-yEmRFP-URA3-CEN-ARS* | This study |
| VSY264 | VSY166 | pVS43 | DSY565 *ura3*::*hph, epa1::EPA1-SAT1-FLIP* | This study |
| VSY265 | VSY264 | - | DSY565 *ura3*::*hph, epa1::EPA1-FRT* | This study |
| VSY266 | VSY265 | pVS20 | DSY565 *ura3*::*hph, epa1::EPA1-FRT, ScPGK1p-URA3-CEN-ARS* | This study |
| VSY267 | VSY265 | pGRB2.3 | DSY565 *ura3*::*hph, epa1::EPA1-FRT, ScPGK1p-yEGFP-URA3-CEN-ARS* | This study |
| VSY268 | VSY265 | pVS19 | DSY565 *ura3*::*hph, epa1::EPA1-FRT, ScPGK1p- yEmRFP-URA3-CEN-ARS* | This study |
| VSY269 | SFY114 | pVS37 | DSY562 *pdr1::PDR1-SAT1*, *epa1::hph-FLIP* | This study |
| VSY270 | SFY115 | pVS37 | DSY562 *pdr1::PDR1^L280F^-SAT1*, *epa1::hph-FLIP* | This study |
| VSY271 | VSY269 | - | DSY562 *pdr1::PDR1-SAT1*, *epa1::FRT* | This study |
| VSY272 | VSY270 | - | DSY562 *pdr1::PDR1^L280F^-SAT1*, *epa1::FRT* | This study |
| VSY273 | VSY190 | pSF53 | DSY2235 *ura3::FRT, pdr1::PDR1^R376W^-SAT1* | This study |
| VSY274 | VSY190 | pSF57 | DSY2235 *ura3::FRT, pdr1::PDR1^T588A^-SAT1* | This study |
| VSY275 | VSY214 | pVS37 | DSY2235 *ura3::FRT, pdr1::PDR1^L280F^-SAT1, epa1::hph-FLIP* | This study |
| VSY276 | VSY275 | - | DSY2235 *ura3::FRT, pdr1::PDR1^L280F^-SAT1, epa1::FRT* | This study |
| VSY277 | VSY273 | pVS20 | DSY2235 *ura3::FRT, pdr1::PDR1^R376W^-SAT1, ScPGK1p-URA3-CEN-ARS* | This study |
| VSY278 | VSY274 | pVS20 | DSY2235 *ura3::FRT, pdr1::PDR1^T588A^-SAT1, ScPGK1p-URA3-CEN-ARS* | This study |
| VSY279 | VSY273 | pGRB2.3 | DSY2235 *ura3::FRT, pdr1::PDR1^R376W^-SAT1, ScPGK1p-yEGFP-URA3-CEN-ARS* | This study |
| VSY280 | VSY274 | pGRB2.3 | DSY2235 *ura3::FRT, pdr1::PDR1^T588A^-SAT1, ScPGK1p-yEGFP-URA3-CEN-ARS* | This study |
| VSY281 | VSY273 | pVS19 | DSY2235 *ura3::FRT, pdr1::PDR1^R376W^-SAT1, ScPGK1p- yEmRFP-URA3-CEN-ARS* | This study |
| VSY282 | VSY274 | pVS19 | DSY2235 *ura3::FRT, pdr1::PDR1^T588A^-SAT1, ScPGK1p- yEmRFP-URA3-CEN-ARS* | This study |
| VSY287 | VSY276 | pVS20 | DSY2235 *ura3::FRT, pdr1::PDR1^L280F^-SAT1, epa1::FRT, ScPGK1p-URA3-CEN-ARS* | This study |
| VSY290 | VSY271 | pVS13 | DSY562 *pdr1::PDR1-SAT1*, *epa1::FRT, ura3::hph* | This study |
| VSY291 | VSY272 | pVS13 | DSY562 *pdr1::PDR1^L280F^-SAT1*, *epa1::FRT, ura3::hph* | This study |
| VSY292 | VSY290 | pVS20 | DSY562 *pdr1::PDR1-SAT1*, *epa1::FRT, ura3::hph, ScPGK1p-URA3-CEN-ARS* | This study |
| VSY293 | VSY291 | pVS20 | DSY562 *pdr1::PDR1^L280F^-SAT1*, *epa1::FRT, ura3::hph, ScPGK1p-URA3-CEN-ARS* | This study |
| VSY294 | VSY290 | pGRB2.3 | DSY562 *pdr1::PDR1-SAT1*, *epa1::FRT, ura3::hph, ScPGK1p-yEGFP-URA3-CEN-ARS* | This study |
| VSY295 | VSY291 | pGRB2.3 | DSY562 *pdr1::PDR1^L280F^-SAT1*, *epa1::FRT, ura3::hph, ScPGK1p-yEGFP-URA3-CEN-ARS* | This study |
| VSY296 | VSY290 | pVS19 | DSY562 *pdr1::PDR1-SAT1*, *epa1::FRT, ura3::hph, ScPGK1p- yEmRFP-URA3-CEN-ARS* | This study |
| VSY297 | VSY291 | pVS19 | DSY562 *pdr1::PDR1^L280F^-SAT1*, *epa1::FRT, ura3::hph, ScPGK1p- yEmRFP-URA3-CEN-ARS* | This study |
| VSY298 | VSY166 | pVS47 | DSY565 *ura3::hph, epa1::(˗600* to *˗527)Δ-EPA1-SAT1-FLIP* | This study |
| VSY299 | VSY298 | - | DSY565 *ura3::hph, epa1::(˗600* to *˗527)Δ-EPA1-FRT* | This study |

**References**

1. **Ferrari S, Ischer F, Calabrese D, Posteraro B, Sanguinetti M, Fadda G, Rohde B, Bauser C, Bader O, Sanglard D.** 2009. Gain of function mutations in *CgPDR1* of *Candida glabrata* not only mediate antifungal resistance but also enhance virulence. PLoS Pathog **5:**e1000268.

2. **Cormack BP, Ghori N, Falkow S.** 1999. An adhesin of the yeast pathogen *Candida glabrata* mediating adherence to human epithelial cells. Science **285:**578-582.

3. **Dujon B, Sherman D, Fischer G, Durrens P, Casaregola S, Lafontaine I, De Montigny J, Marck C, Neuveglise C, Talla E, Goffard N, Frangeul L, Aigle M, Anthouard V, Babour A, Barbe V, Barnay S, Blanchin S, Beckerich J, Beyne E, Bleykasten C, Boisrame A, Boyer J, Cattolico L, Confanioleri F, De Daruvar A, Despons L, Fabre E, Fairhead C, Ferry-Dumazet H, Groppi A, Hantraye F, Hennequin C, Jauniaux N, Joyet P, Kachouri R, Kerrest A, Koszul R, Lemaire M, Lesur I, Ma L, Muller H, Nicaud J, Nikolski M, Oztas S, Ozier-Kalogeropoulos O, Pellenz S, Potier S, Richard G, Straub M, Suleau A, Swennen D, Tekaia F, Wesolowski-Louvel M, Westhof E, Wirth B, Zeniou-Meyer M, Zivanovic I, Bolotin-Fukuhara M, Thierry A, Bouchier C, Caudron B, Scarpelli C, Gaillardin C, Weissenbach J, Wincker P, Souciet J.** 2004. Genome evolution in yeasts. Nature **430:**35-44.

4. **Vale-Silva L, Ischer F, Leibundgut-Landmann S, Sanglard D.** 2013. Gain-of-function mutations in *PDR1*, a regulator of antifungal drug resistance in *Candida glabrata*, control adherence to host cells. Infect Immun **81:**1709-1720.
